# *In muro* deacetylation of xylan affects lignin properties and improves saccharification of aspen wood

**DOI:** 10.1186/s13068-017-0782-4

**Published:** 2017-04-20

**Authors:** Prashant Mohan-Anupama Pawar, Marta Derba-Maceluch, Sun-Li Chong, Madhavi Latha Gandla, Shamrat Shafiul Bashar, Tobias Sparrman, Patrik Ahvenainen, Mattias Hedenström, Merve Özparpucu, Markus Rüggeberg, Ritva Serimaa, Martin Lawoko, Maija Tenkanen, Leif J. Jönsson, Ewa J. Mellerowicz

**Affiliations:** 1 Department of Forest Genetics and Plant Physiology, Swedish University of Agricultural Sciences, S-901 83 Umeå, Sweden; 2 Department of Food and Environmental Sciences, University of Helsinki, P.O. Box 27, 00014 Helsinki, Finland; 3 Department of Chemistry, Umeå University, S-901 87 Umeå, Sweden; 4 Department of Physics, University of Helsinki, P O Box. 64, 00014 Helsinki, Finland; 5 Institute for Building Materials, Swiss Federal Institute of Technology (ETH Zürich), 8093 Zurich, Switzerland; 6 Laboratory of Applied Wood Materials, Empa, Dübendorf, 8600 Dübendorf, Switzerland; 7 Department of Fiber and Polymer Technology, Wallenberg Wood Science Center, WWSC, Royal Institute of Technology, KTH, SE-100 44 Stockholm, Sweden; 8 Department of Biochemistry, Purdue University, West Lafayette, IN 47907-2063 USA; 9 Department of Biology and Biological Engineering, Division of Industrial Biotechnology, Chalmers University of Technology, Kemivägen 10, SE-412 96 Göteborg, Sweden

**Keywords:** Acetylation, Xylan, Saccharification, Wood, *Populus*

## Abstract

**Background:**

Lignocellulose from fast growing hardwood species is a preferred source of polysaccharides for advanced biofuels and “green” chemicals. However, the extensive acetylation of hardwood xylan hinders lignocellulose saccharification by obstructing enzymatic xylan hydrolysis and causing inhibitory acetic acid concentrations during microbial sugar fermentation. To optimize lignocellulose for cost-effective saccharification and biofuel production, an acetyl xylan esterase *An*AXE1 from *Aspergillus niger* was introduced into aspen and targeted to cell walls.

**Results:**

*An*AXE1-expressing plants exhibited reduced xylan acetylation and grew normally. Without pretreatment, their lignocellulose yielded over 25% more glucose per unit mass of wood (dry weight) than wild-type plants. Glucose yields were less improved (+7%) after acid pretreatment, which hydrolyses xylan. The results indicate that *An*AXE1 expression also reduced the molecular weight of xylan, and xylan–lignin complexes and/or lignin co-extracted with xylan, increased cellulose crystallinity, altered the lignin composition, reducing its syringyl to guaiacyl ratio, and increased lignin solubility in dioxane and hot water. Lignin-associated carbohydrates became enriched in xylose residues, indicating a higher content of xylo-oligosaccharides.

**Conclusions:**

This work revealed several changes in plant cell walls caused by deacetylation of xylan. We propose that deacetylated xylan is partially hydrolyzed in the cell walls, liberating xylo-oligosaccharides and their associated lignin oligomers from the cell wall network. Deacetylating xylan thus not only increases its susceptibility to hydrolytic enzymes during saccharification but also changes the cell wall architecture, increasing the extractability of lignin and xylan and facilitating saccharification.

**Electronic supplementary material:**

The online version of this article (doi:10.1186/s13068-017-0782-4) contains supplementary material, which is available to authorized users.

## Background

Woody biomass is a major source of renewable energy, which is needed to meet the global demand for electricity, heat, and clean liquid fuels [1]. The productivity of hardwood tree species such as eucalypts, poplars, aspens, and willows can exceed 40 m^3^ ha^−1^ year^−1^ and several genetic approaches can be used to modify their wood to facilitate cost-effective biorefining.

Hardwood lignocellulose is a composite of heterogeneous polymers, including crystalline cellulose, xylans, and lignins, which are attached to each other by covalent and non-covalent linkages involving both their backbones and side groups. Understanding these interactions is essential for designing efficient methods for lignocellulose deployment. Acetyl side groups are mainly present on glucuronoxylan [2, 3], where 40–70% of xylopyranosyl (Xyl*p*) units are acetylated at the C2 and/or C3 positions [4–8]. Acetyl substitution hinders enzymatic glucuronoxylan hydrolysis [9, 10], and because glucuronoxylan coats cellulose microfibrils [6], its efficient hydrolysis is necessary for cellulose conversion [11]. In biorefineries, most glucuronoxylan is removed during pretreatment and its acetyl groups are converted into acetic acid—a potent inhibitor of microorganisms such as *Saccharomyces cerevisiae* that are needed for subsequent fermentation [12, 13]. Reducing glucuronoxylan acetylation could therefore improve the conversion of biomass into biofuels. However, saccharification of Arabidopsis mutants with reduced glucuronoxylan acetylation has not improved sugar yields [14–16]. Moreover, many of these mutants exhibited dwarfism, mechanically weak stems, and collapsed xylem vessels [14–18], indicating that glucuronoxylan acetylation has important but poorly understood biological functions. These effects were avoided when glucuronoxylan was deacetylated *in muro* by *Aspergillus nidulans* acetyl xylan esterase [19]. Lignocellulose from Arabidopsis plants expressing *Aspergillus niger* acetyl xylan esterase 1 (*An*AXE1] yielded more sugars and ethanol [20] than that from wild-type plants. Encouraged by these results, we have generated aspens expressing *An*AXE1 to study its effects on lignocellulose properties relevant to saccharification. The enzyme facilitated lignocellulose saccharification by reducing its acetyl content and by inducing unanticipated effects on the cell walls *in planta* that increased lignin extractability. These findings will be relevant for all applications of lignocellulose involving lignin extraction.

## Results

### Expressing acetyl xylan esterase in hybrid aspen did not affect plant growth

**Fig. 1:**
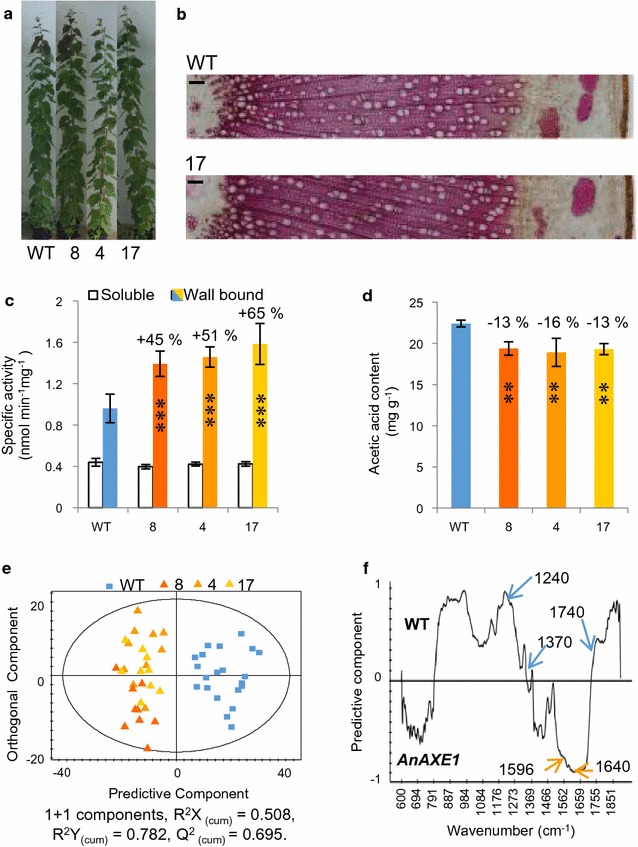
*An*AXE1 overexpressing aspen exhibits normal growth and reduced cell wall acetyl content. **a** Appearance of 2-month-old trees of transgenic lines 8, 4, and 17 compared to WT. **b** Stem cross sections stained with phloroglucinol from representative line 17 and WT trees. *Scale Bar* 100 μm. **c** Acetyl esterase-specific activity observed in soluble and wall-bound protein fractions extracted from developing wood with *p*-naphthyl acetate used as substrate. **d** Cell wall acetyl content determined by acetic acid release after saponification. Data in **c** and **d** are mean ± SE, *n* = 5 biological replicates. *Asterisks* mark lines significantly different from WT: **P ≤ 0.05; ***P ≤ 0.01 (Student’s *t* test). **e** OPLS-DA scatter plot of FT-IR data obtained for wood powder from transgenic and WT trees. **f** Corresponding loading plot, showing variables that contributed to the separation. Signals assigned to acetyl esters (1240, 1370, and 1740), indicated by *blue arrows*, were more intense in the WT; signals assigned to absorbed water and lignin (1640 and 1596, respectively), indicated by *orange arrows*, were more intense in transgenic trees

*35S::AnAXE1* [20, 21] was expressed in hybrid aspen (*Populus tremula* L. *x tremuloides* Michx.). From 20 independent lines screened for high transgene expression, three lines with similarly high transcript levels (Fig. S1A) were selected and grown in the greenhouse. Transgenic plants exhibited no morphological alterations (Fig. 1a) and their growth parameters (height, internode length, and diameter), and wood mechanical properties measured by micromechanical axial stretching (E-modulus and ultimate stress) were unchanged (Additional file 1) compared to the wild type (WT). The acetyl esterase activity of wall-bound protein extracts from the transgenic plants’ developing wood tissues was 45–65% higher than that of the WT when tested against the synthetic substrate *para*-nitrophenyl acetate, but soluble protein extracts from transgenic and WT plants exhibited similar levels of esterase activity (Fig. 1c). These data indicate that *An*AXE1 is active in cell walls and does not affect aspen growth or development, as in Arabidopsis [20].

### *AnAXE1*-expressing trees exhibited reduced xylem *O*-acetylation and reduced xylan acetylation at the C-2 position

To determine whether the cell wall acetyl content was reduced in transgenic plants, the acetic acid released from their wood after saponification was quantified, revealing a reduction of 13–16% (Fig. 1d). Furthermore, wood powder analyses by Fourier transform infrared spectroscopy (FT-IR) revealed a clear separation between WT and transgenic samples (Fig. 1e). The signals that contributed to the separation were assigned to acetyl esters (1370, 1240, and 1740 cm^−1^), absorbed water (1640 cm^−1^), and lignin (1596 cm^−1^) [20, 22]. Signals assigned to acetyl ester were less intense in the spectra of the transgenic samples than in the WT spectra; the opposite was true for the signals assigned to lignin and absorbed water (Fig. 1f). Together, these results show that, compared to WT plants, *An*AXE1-expressing trees have decreased acetylation, and suggest that they have greater levels of absorbed water, and a different lignin content or lignin structure.

**Fig. 2:**
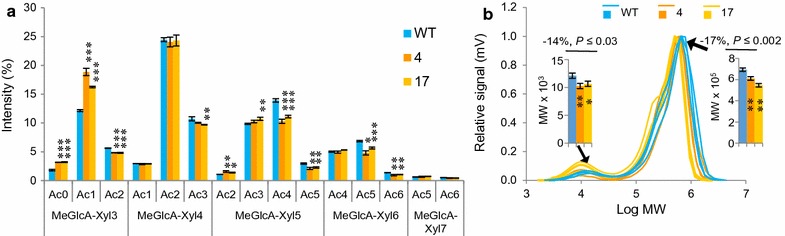
Xylan analysis in lines expressing *AnAXE1* revealed reduction of *O*-acetylation and chain length. **a** OLIMP analysis of acidic xylo-oligosaccharides (XOS) released by endoxylanase. Acidic XOS ranged from DP 3 to 7 (MeGlcA-Xyl3 to MeGlcA-Xyl7) and had 0-6 acetyl groups (Ac0–Ac6). Mean ± *SE*, *n* = 2 replicates representing pools of 5 individual trees. **b** Size exclusion chromatography indicating that transgenic lines have smaller polymer size of xylan and of xylan–lignin complexes (and/or lignin co-extracted with xylan) than the WT. Profiles of three samples are shown per line. The *insets* show differences among *lines* in the molecular weight (MW) of the smaller peak, corresponding to xylan, and the main peak, corresponding to xylan, xylan–lignin complexes and lignin co-extracted with xylan. Data and mean ± SE, *n* = 3–5 biological replicates. **a**, **b** *Asterisks* indicate *lines* differing significantly from WT: *P ≤ 0.1, **P ≤ 0.05; ***P ≤ 0.01 (Student’s *t* test). *P* values shown above the *bars* correspond to post-ANOVA contrast analysis comparing both transgenic lines to WT

To investigate how *An*AXE1 affected glucuronoxylan acetylation, heat-treated alcohol-insoluble cell wall residues were digested with a GH10 *endo*-1,4-β-xylanase, and the released acidic and neutral xylo-oligosaccharides (XOS) were analyzed by oligo mass profiling (OLIMP). XOS with degree of polymerization (DP) values ranging from DP3 to DP7 with between 0 and 6 acetyl groups were detected in the acidic fractions (Fig. 2a), and the distributions for all DPs (except DP7, whose abundance was low) revealed shifts to lower acetylation in the transgenic samples. There was also a clear shift from DP5 and DP6 to DP3 among the acidic XOS from transgenic lines, indicating that the glucuronoxylan in the transgenic samples was more accessible to GH10 xylanase. A similar shift from high to low XOS acetylation was observed for the neutral XOS (Additional file 2). This indicates that *An*AXE1 deacetylates both neutral and MeGlcA-substituted regions of the xylan backbone *in planta*.

2D NMR spectroscopy of DMSO-extracted xylan indicated that 35% of the Xyl*p* residues were acetylated in the WT (Additional file 2). This was within a range reported for hardwood xylan [7, 23]. Most of the Xyl*p* residues were monoacetylated at C-2 (22%), but some were monoacetylated at C-3 (3%), di-acetylated (3%) or had both (Me)GlcA and acetyl residues (6%) (Additional file 2). No glucuronate-substituted residues without acetyl groups were detected. Transgenic lines exhibited approximately 10% less monoacetylation at C-2. This suggests that *An*AXE1 preferentially acts at the C-2 position in aspen wood, in keeping with the observed in vitro positional specificities of other CE1 enzymes [24]. These results could be affected by acetyl migration [25] and should be considered relative, but they support the suggested lower level of xylan acetylation in transgenic lines.

### Lignocellulose from transgenic trees yielded more sugars during enzymatic saccharification

**Fig. 3:**
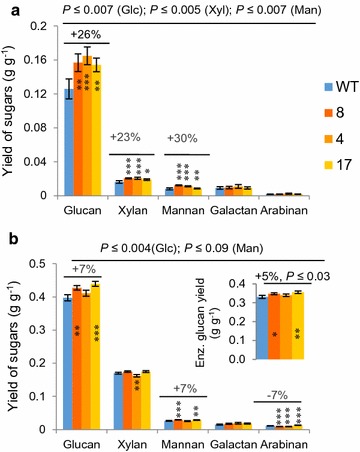
Transgenic trees expressing *AnAXE1* had higher sugar yields. **a** Sugar yields after enzymatic hydrolysis without pretreatment. **b** Combined sugar yields after acid pretreatment and enzymatic hydrolysis. Yields are expressed per unit wood dry weight. The *inset* in **b** represents glucan yields for enzymatic hydrolysis only. Mean ± SE, *n* = 5 biological replicates. *Asterisks* in **a** and **b** mark lines significantly different from WT: *P ≤ 0.1; **P ≤ 0.05; ***P ≤ 0.01 (Student’s *t* test). *P* values shown above the *bars* correspond to post-ANOVA contrast analysis comparing all transgenic lines to WT

Because removing acetyl groups from aspen lignocellulose before enzymatic saccharification improves Glc and Xyl yields [26, 27], we measured the sugar yields after enzymatic hydrolysis obtained from the lines with and without reduced acetylation. Enzymatic hydrolysis without pretreatment converted approx. 27% of available Glc in the WT but was 24% higher in lines 4 and 17 (*P* ≤ 5%), suggesting increased accessibility of Glc in these transgenic lines. The wood of transgenic lines released 23–30% more sugars per unit weight than the WT (Fig. 3a).

In the WT, enzymatic saccharification after acid pretreatment converted approximately 85% of the available Glc. When applied to the transgenic lines, the combined yields of acid and enzymatic hydrolysis for Glc and Man were 7% higher than for the WT, but that of Xyl (which is predominantly liberated during acid hydrolysis) was unchanged (Fig. 3b). The greater Glc yields from the transgenic lines were due to more efficient acidic (Additional file 3), and enzymatic hydrolysis (Fig. 3b inset). The overall Glc conversion for lines 4 and 17 was 5% greater on average than for the WT (*P* ≤ 0.04). This shows that the introduced xylan modification increased Glc yields even for saccharification with acid pretreatment, which achieves almost complete Glc conversion. However, the improvement was less pronounced than in the case of saccharification without pretreatment.

### *AnAXE1* affected the cell wall’s polysaccharide and lignin composition

To determine whether *in muro* cell wall deacetylation affected the cell wall composition, the contents of extractives, hemicellulose, cellulose, and lignin in transgenic and WT wood samples were analyzed (Additional file 4). No differences were detected other than a small increase in cellulose in line 4, which was not confirmed by a subsequent Updegraff cellulose content measurement (Additional file 5). Conversely, structural studies of cellulose using NMR revealed greater crystallinity in the transgenic lines (Additional file 5). Sample crystallinity determined by X-ray scattering showed a similar trend.

Monosaccharide composition analysis of non-cellulosic polymers by acid methanolysis showed a relative decrease in the abundance of Xyl and increases in that of Glc and Gal in transgenic lines (Additional file 5). To determine whether the lower Xyl content in *An*AXE1-expressing lines might be due to their shorter xylan chains, the hemicelluloses were alkali-extracted from extractives- and pectin-free wood, and their polymeric fraction was analyzed by size exclusion chromatography using the pulsed amperometric detector (PAD). The size distribution profiles (Fig. 2b) featured a predominant peak in the high (≈600,000) molecular weight (MW) region and a much smaller peak in the low (≈10,000) MW region. The low MW peak was within a range previously reported for hardwood xylan, but the high MW peak corresponded to much greater values [23]. To exclude a possibility that the samples were contaminated with cellulose, we analyzed sugars in the hydrolyzed samples detecting only Xyl, which indicates the absence of polysaccharides other than xylan in the samples. To determine if lignin was contaminating the samples, the UV detector was used in tandem with PAD (Additional file 6). The UV signals were detected with the major peak predominantly overlapping with the main peak detected by PAD and a few smaller peaks, none of which clearly overlapping with the low MW PAD peak. This indicates that the main peak detected by PAD included lignin co-extracted with xylan, as it has previously been observed in aspen [7]. Interestingly, both high and low MW peaks were shifted towards 17 and 14% lower MW, respectively, in the transgenic samples (insets in Fig. 2b). Similar shifts were observed in the main UV peaks (Additional file 6). These results indicate that the xylan chain length and the size of xylan–lignin polymer (and/or lignin polymer that was co-extracted with xylan) were reduced in *An*AXE1 expressing lines.

**Fig. 4:**
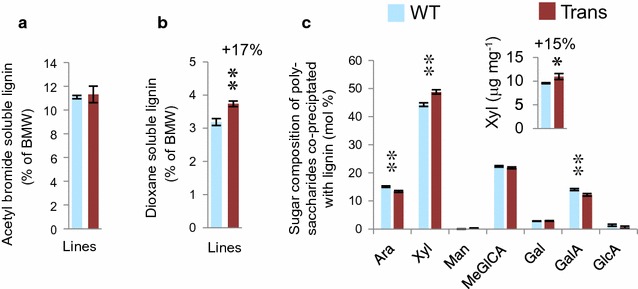
Transgenic trees had similar lignin content but different lignin solubility and associated sugars compared to WT. **a** Acetyl bromide-soluble and **b** dioxane-soluble lignin contents in ball milled wood (BMW). **c** Composition of sugars in polysaccharides co-precipitated with lignin. Lignin was extracted from BMW with acidified dioxane as described in “Methods”, and precipitated with water. The inset shows the absolute change in the Xyl content of the lignin precipitate. Data in **a** and **c** are mean ± SE, *n* = 3 replicates representing pooled samples of six transgenic (Trans; two trees from each lines, 8, 4, 17), or wild-type (WT) trees. *Asterisks* indicate means significantly different from WT, *P ≤ 0.1; **P ≤ 0.05 (ANOVA)

Cell wall compositional analysis by pyrolysis–GC–MS revealed no changes in the relative abundance of lignin or carbohydrates in the transgenic lines but did indicate a reduced syringyl to guaiacyl (S/G) ratio (Additional file 7). Lignin was further analyzed in ball milled wood (BMW). The acetyl bromide-soluble lignin contents of BMW from transgenic and WT samples were identical (Fig. 4a). However, the content of dioxane-soluble lignin, which constitutes approx. 30% of all lignin and is the native lignin fraction associated with polysaccharides [28, 29], was 17% higher in the transgenic lines (Fig. 4b). Thus, although the overall lignin content was unchanged in *An*AXE1 expressing lines, their lignin composition and solubility were significantly altered.

To identify mechanisms potentially responsible for the observed changes in xylan chain length and lignin S/G ratio, we analyzed the mRNA expression of key proteins in the xylan backbone synthase complex, GT43A (Potri.006G131000) and GT43B (Potri.016G086400), and S-lignin biosynthesis pathway, F5H (Potri.007G016400), and COMT (Potri.012G006400). None of these transcripts was significantly affected in the transgenic lines (Additional file 8), indicating that the observed alterations in xylan and lignin are governed by other factors.

### Analysis of different lignin–carbohydrate complexes (LCCs) fractions

To explain the increased extractability and digestibility of cell wall polymers in trees expressing *An*AXE1, we analyzed different fractions of the lignin–carbohydrate complexes (LCCs) in transgenic and WT samples. Lignin extracted with acidified dioxane was precipitated to yield the native lignin fraction with associated polysaccharides [28, 29]. This precipitate contained 15% more Xyl in transgenic than in WT samples (Fig. 4c inset). Moreover, its monosaccharide composition (analyzed excluding Glc to avoid possible contributions from starch and to show changes in matrix polysaccharides) revealed increased levels of Xyl and lower levels of Ara and GalA (Fig. 4c). These data indicate that the carbohydrates co-precipitated with native lignin are enriched in xylan but have fewer pectins in transgenic samples.

**Fig. 5:**
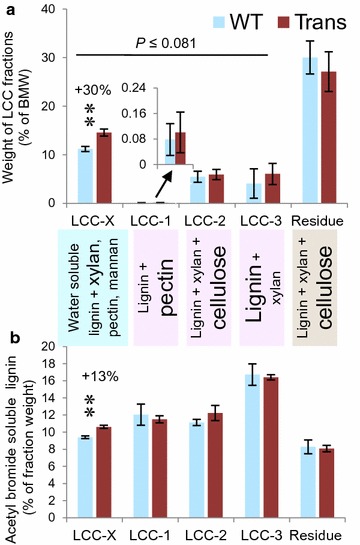
Analysis of lignin–carbohydrate complexes (LCC) in transgenic and WT trees. Ball milled wood (BMW) from WT and transgenic lines 8, 4, and 17 was fractionated as described in “Methods”. **a** Weights of different LCC fractions. Mean ± SE, data from two experiments. *P* values above the line correspond to the probability that the higher summed LCC mass in transgenic lines is due to chance (ANOVA). The compounds in the LCC fractions were deduced from sugar, cellulose (Additional file 9), and lignin analyses, and the most abundant compounds are indicated by large font. **b** Acetyl bromide-soluble lignin content in each LCC fraction. Mean ± SE, *n* = 3, as in Fig. 4. **Significantly different means in transgenic lines at *P* ≤ 0.05 (ANOVA)

Next, BMW samples were sequentially extracted and fractionated to obtain lignin fractions associated with different carbohydrates [30]. Sugar composition, Updegraff cellulose content (Additional file 9), and acetyl bromide-soluble lignin (Fig. 5b) analyses of these fractions indicated that the LCC-X fraction contained water-soluble lignin, xylan, mannan and pectin, LCC-1 contained predominantly homogalacturonan and lignin, LCC-2 contained xylan, cellulose and lignin, LCC-3 contained mostly lignin with some xylan, glucomannan and pectin, and the residue included the insoluble cellulose-xylan fraction along with some lignin. LCC-X was the most abundant extracted fraction (approx. 10% of BMW in WT), and it was significantly more abundant in transgenic samples (Fig. 5a). Moreover, its lignin content in the transgenic samples was greater than in the WT (Fig. 5b). Because of the higher yield of this fraction from the transgenic samples and its higher lignin content, the transgenic samples yielded approx. 38% more lignin in water extracts per weight of wood than the WT.

Further pyrolysis–GC–MS analysis of the LCC fractions indicated that the water-soluble lignin from the transgenic lines had a greater proportion of G-units than that from the WT (Additional file 10). The relative abundance of lignin pyrolysis products and Xyl were also increased in the LCC-1 fractions from transgenic samples (Additional files 9, 10). These results suggest that more lignin and xylan are extracted along with pectins from the transgenic samples than the WT. Interestingly, whereas the wood from the transgenic samples and most of its fractions had lower S/G ratios than their WT counterparts (Additional files 7, 10), the opposite was true for the LCC-1 fraction, indicating that the pectin-associated lignin in transgenic samples was particularly enriched in S lignin.

These analyses showed that *in muro* enzymatic xylan, deacetylation changed the extractabilities of pectins, xylan, and lignin, possibly by changing their associations with one-another. Importantly, deacetylation increased the lignin and xylan extractability of the transgenic lignocellulose.

## Discussion

We have shown that partial xylan deacetylation by the acetyl xylan esterase *An*AXE1 expressed *in planta* and targeted to cell walls substantially increased (~26%) the Glc yields of enzymatic saccharification without pretreatment and modestly (~7%) increased yields after acid pretreatment (Fig. 3). Similar results were obtained in transgenic aspen with reduced expression of reduced wall acetylation (RWA) genes, which had reduced xylan acetylation [31]. These reports are consistent with the increased rate of sugar production in Arabidopsis expressing *An*AXE1 during saccharification after hot water pretreatment, and the negligible improvement observed after acid pretreatment [20]. Similarly, no increase in saccharification after acid pretreatment was observed when a closely related acetyl xylan esterase was expressed in Arabidopsis [19]. Acid pretreatment thus seems to nullify the benefits of xylan deacetylation. Surprisingly, however, acetyl xylan esterase expression substantially improved saccharification yields after alkali pretreatment [20]. This strongly suggests that the benefits of xylan deacetylation stem from changes in cell wall properties rather than the deacetylation per se.

**Fig. 6:**
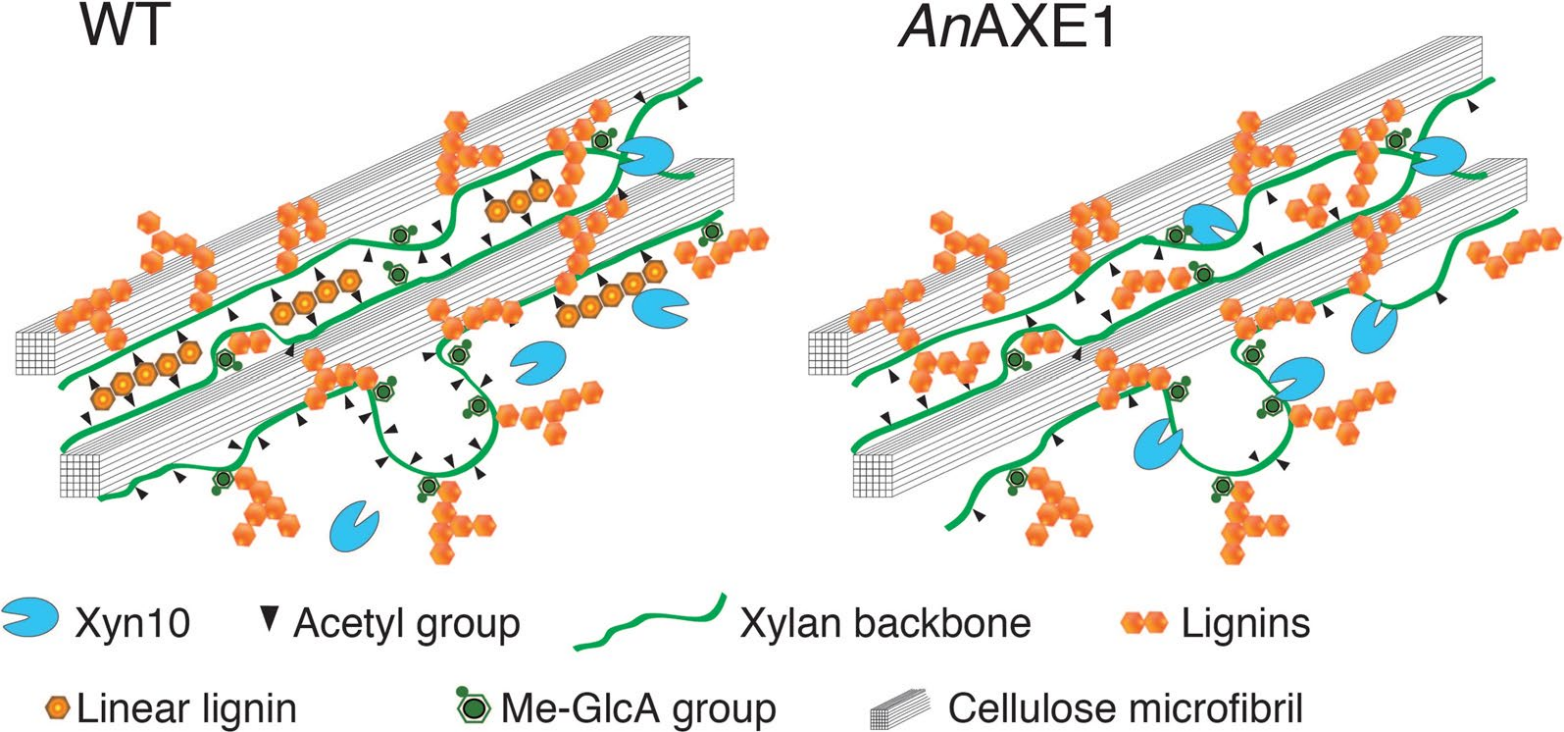
Hypothetical model proposing how *An*AXE1 could affect lignin and xylan solubility in transgenic lines. Deacetylation of xylan is hypothesized to render it more susceptible to cell wall-resident family GH10 transglycosylases such as *Ptxt*XYN10A [32] and endoxylanases. GH10 enzymes do not tolerate substitutions at subsite −1 due to steric hindrance [39], and therefore, xylan domains with acetylation at consecutive Xyl*p* units are expected to be protected from these enzymes. As found in [5, 6], the majority of xylan present in secondary walls has acetylation on alternate Xyl*p* units, and such xylan domains have been proposed to interact with hydrophilic side of cellulose microfibrils [6]. Only minor xylan domains have acetylation on consecutive Xyl*p* [5, 6], and it was reasoned that such acetylation pattern precludes the binding to cellulose [6]. However, these regions are expectedly available for interactions with lignin, for example, via MeGlcA side chains. Deacetylation of these minor domains would have rendered them even more prone to interaction with lignin via newly available OH groups on Xyl*p* as proposed in [33], and to hydrolysis by GH10, as discussed above. Consequently, these domains along with their associated lignin polymers would be liberated from the cellulose-xylan network in *An*AXE1 expressing *lines*

This work identified at least three changes in cell walls caused by *in planta* deacetylation that could have benefited saccharification: a decrease in xylan chain length, a decrease in MW of xylan–lignin complex and/or lignin co-extracted with xylan, and an increase in lignin solubility. The unchanged expression of genes crucial for biosynthesis of the xylan backbone in the transgenic plants (Additional file 8) strongly suggests that the decrease in xylan chain length is a direct consequence of deacetylation rather than an indirect effect involving modification of xylan biosynthesis. We propose that deacetylated xylan is more susceptible to endogenous, wall-residing hydrolases, and transglycosylases such as *Ptxt*Xyn10A [32], which generate XOS, some of which would be covalently linked to lignin and solubilized as xylan–lignin complexes, as illustrated in Fig. 6. GH10 activity would thus oligomerize deacetylated xylan and liberate the associated lignin from the cell wall network. Moreover, it has recently been shown that less acetylated xylan may have a higher prevalence of ether and glycosidic bonds to lignin [33], implying that more lignin could be associated with the GH10 oligomerized xylan in the *An*AXE1 plants. The proposed mechanism (Fig. 6) is supported by four observations: (1) the increased solubility of lignin in dioxane-water in transgenic plants (Fig. 4b), (2) the increased water solubility of lignin (Fig. 5), (3) the reduced cell wall Xyl content (Additional file 5) and xylan chain length (Fig. 2b), and (4) the increased Xyl content in sugars co-precipitated with pectic LCCs (Additional file 9) and lignin (Fig. 4c). These data support the notion that xylan deacetylation *in planta* facilitates solubilisation of a fraction of lignin with associated xylan. Moreover, we observed that the xylan–lignin fraction had a lower MW in *An*AXE1 expressing lines (Fig. 2b; Additional file 6), supporting the increased lignin solubility. Because acid pretreatment hydrolyzes xylan, including its lignin-associated domains, *An*AXE1 has minimal effects on saccharification after acid pretreatment [19, 20] (Fig. 3b).

*An*AXE1 also increased cellulose crystallinity (Additional file 5), which would adversely affect saccharification [27]. Interestingly, a similar increase in cellulose crystallinity following xylan deacetylation was observed during saccharification in vitro [26], suggesting that this process is spontaneous, perhaps, resulting from reduced xylan coating of microfibrils, causing them to merge into macrofibrils.

Another difference between *AnAXE1*-expressing and WT lines is that the former have a lower S/G ratio but the same lignin content. The mechanism causing this change is unknown but may be related to lignin polymerization rather than monolignol biosynthesis, because the S-monolignol biosynthetic genes *F5H* and *COMT* exhibited no transcriptional changes (Additional file 8). S-lignin monomer formation may be favored by the properties of the spaces between acetylated xylan-coated microfibrils (Fig. 6). The lower S/G ratio may have been partly responsible for the changes in saccharification yields, but it is not yet clear how the S/G ratio affects saccharification in poplar [34, 35].

Saccharification could also benefit from deacetylation of other than xylan polymers, which probably occur in *An*AXE1-expressing plants considering the known broad specificity of CE1 esterases [3, 21]. Indeed, the previous results in Arabidopsis suggested that *An*AXE1 may deacetylate xyloglucan [20]. Although other acetylated polysaccharides are less abundant than xylan in hardwoods, one cannot rule out a possibility that their deacetylation also plays a role in reduced recalcitrance of transgenic lines.

As in Arabidopsis [20], xylan deacetylation by *An*AXE1 in cell walls did not affect growth and morphology in hybrid aspen. Conversely, reducing xylan biosynthesis in the cell walls of Arabidopsis mutants caused severe defects [14–18] including irregular xylem phenotypes and abnormal plant development. The defects in xylem morphology were reversed in the *esk1* mutant overexpressing the *At*GUX1 enzyme that adds glucuronate decorations to the xylan backbone, indicating that a branched xylan structure is essential for secondary wall biosynthesis [36]. However, glucuronation necessitates the use of α-glucuronidases for xylan hydrolysis. Therefore, expressing microbial enzymes that deacetylate xylan post-synthesis may be a better strategy for tailoring biomass for saccharification. Such engineered plants would also provide biomass with a greater fermentation potential for bioethanol production [20].

## Conclusions

Hardwoods have enormous potential as renewable sources of sugars because of their high contents of carbon-rich polysaccharides, but the conversion of these polysaccharides to sugars (saccharification) is hampered by xylan acetylation, which shields the xylans and associated cellulose from hydrolytic enzymes. We found that introducing a fungal gene encoding an acetyl xylan esterase into aspen, and targeting it to cell walls, is a promising strategy for improving the economic viability of hardwood biorefining. Such engineered plants grow well and their wood yields more sugars per unit weight than the wild type. Moreover, we identified a mechanism by which xylan deacetylation exerts its positive effects on saccharification: compared to the wild type, the engineered plants exhibit shorter xylan chain lengths and greater lignin extractability. This changes the architecture of the cell walls without compromising their mechanical properties. These results provide new insights into the role of xylan acetylation in cell walls.

## Methods

### Generation of transgenic aspen

A construct expressing cDNA encoding *AnAXE1* (CAA01634) under the control of the *35S* promoter was cloned into the pEntry D-TOPO vector using *aaacccaattacaac*CATGTCGCCAAGCGCAGTGGTAGCCTCCAA and *tca*AGCAAACCCAAACCACTCCATATCCTTATC as the forward and reverse primers, and subcloned into the binary vector pK2GW7 (Gateway^®^ System; Life Technologies™, Stockholm, Sweden). This vector was transformed into Agrobacterium (GV3101) and used to transform hybrid aspen (*Populus tremula* L*.x tremuloides* Michx., clone *T89*) as previously described [22].

*Transcript level* Total RNA was extracted from developing wood and cDNA was synthesized using iScript™ (Bio-Rad, Hercules, CA, USA). PCR was run on the Light Cycler 480 II (Roche, Rotkreuz, Switzerland) using SYBR Green (Bio-Rad) and the primers listed in Additional file 11. Relative expression was calculated by the ∆∆Ct method, and expressed as a fold change [22].

*Acetyl esterase activity* Soluble and wall-bound proteins were extracted according to [32] from developing wood, and acetyl esterase activity was determined in extracts using 4-nitrophenyl acetate as the substrate [20]. One unit of enzyme is the amount of enzyme needed to release 1 nmol of 4-nitrophenol per min at 37 °C.

### Wood cell wall analyses

Wood from internodes 44–60 was freeze-dried, ground to 0.5 mm particles (rough wood powder), and then ball milled to fine wood powder [37] or milled in a planetary ball mill PM400 (Retsch) for 9 h to obtain ball milled wood (BMW). The fine wood powder was analyzed by FT-IR spectroscopy and GC–MS pyrolysis as previously described [37]. Data were analyzed using SIMCA-P (version 11.0.0.0, Umetrics AB, Sweden).

Fine wood powder was sequentially extracted and fractionated into extractives, hemicelluloses, cellulose, and lignin [37]. Cellulose content and the monosaccharide composition of non-cellulosic polysaccharides were determined in extractives-free fine wood powder by Updegraff’s method and acid methanolysis, respectively [37]. Cellulose crystallinity was measured by NMR and sample crystallinity was measured by wide angle X-ray scattering, as described in the Additional file 12. Acetyl content was determined after saponification of fine wood powder using K-ACET (Megazyme, Wicklow, Ireland). Acetyl bromide-soluble lignin was determined according to [38].

### Xylan analyses

*OLIMP* Xylan acetylation patterns were carried out as previously described [5] with modifications detailed in Additional file 12.

*NMR* Acetylated xylan was extracted from alcohol-insoluble residue and NMR spectra were acquired using a Bruker Avance III HD 850 MHz spectrometer to obtain 2D 1H-13C HSQC spectra as described in Additional file 12.

*Size exclusion chromatography* Polymeric xylan was extracted from extractives-free, depectinized fine wood powder as described in Additional file 12, and analyzed by size exclusion chromatography with a pulsed amperometric detector in tandem with a UV detector at MoRe Research (Örnsköldsvik, Sweden).

### Pretreatment and saccharification of wood

Rough wood powder was fractionated and processed as described in Additional file 12. Acid pretreatment was performed with 1% sulphuric acid (w/w) using a single-mode microwave system (Initiator Exp, Biotage, Uppsala, Sweden) at 165 °C for 10 min. Enzymatic hydrolysis was performed at 45 °C for 72 h with 50 mg of a 1:1 (w/w) mixture of Cellulase 1.5 L (a cellulase-rich liquid enzyme preparation from *Trichoderma reesei* ATCC 26921 with a stated activity of 700 endoglucanase units per g) and Novozyme 188 (a cellobiase-rich liquid enzyme preparation from *A. niger* with a stated activity of 250 cellobiase units per g (both from Sigma Aldrich). The liquid fractions were analyzed using high-performance anion-exchange chromatography (HPAEC), as previously described [37].

### Analysis of LCCs

The LCC fractionation procedure was modified from [30] and is described in detail in Additional file 12. Briefly, BMW was treated with water at 60 °C for 3 h and the supernatant was freeze-dried to give LCC-X. The pellet was digested with Fibercare containing a mixture of endoglucanases (gift from Novozymes). The resulting supernatant was precipitated with 5% Ba(OH)_2_, and the precipitate was dissolved in glacial acetic acid, re-precipitated in 4 volumes of absolute ethanol, re-dissolved in a small amount of water, dialyzed in a Cellulose RC dialysis membrane (1000 MWCO, Spectrum Labs, Rancho Dominguez, USA) against water, and freeze-dried to yield LCC-1. The pellet was dissolved in DMSO at 60 °C, the residue was saved, and the supernatant was precipitated with 1.25% (w/v) NaCl. The precipitate was dissolved in water, dialyzed as above, and freeze-dried to yield LCC-2. The supernatant after NaCl precipitation was precipitated with four volumes of ethanol and the precipitate was dissolved in water, dialyzed as above, and freeze-dried to yield LCC-3. Dioxane-water-soluble lignin was extracted with a dioxane:water mixture (96:4; v:v) from BMW for 48 h and quantified by absorbance at 280 nm assuming an absorptivity of 18.21 [Lg^−1^ cm^−1^] [38]. Acidic dioxane-soluble lignin was extracted with a dioxane:water mixture (96:4; v/v) supplemented with 0.66 mL 12 M HCl for 2 h at 100 °C, precipitated O/N with water as described in Additional file 12, and freeze-dried.

### Statistical analysis

Data were analyzed using the JMP^®^ Pro 12.0.1 program (www.jmp.com) by the analysis of variance (ANOVA) followed (when needed) by post hoc tests: 1) the *t* test, to compare each individual transgenic lines with WT, and 2) the contrast analysis, to compare an average response of all transgenic lines with WT. The samples were independent, and we have assumed that the variables had normal distributions.

## Additional files

**Additional file 1.** Transgene expression levels and morphological and mechanical parameters of transgenic trees.

**Additional file 2.** Transgenic trees have reduced acetylation of xylan.

**Additional file 3.** Yield of sugars in pretreatment liquid of transgenic and WT samples.

**Additional file 4.** Contents of wood components in transgenic trees determined by sequential extractions and weighing.

**Additional file 5.** Changes in polysaccharide composition and crystallinity in transgenic trees.

**Additional file 6.** Size exclusion chromatography of xylan extracted from wood using UV and PAD detectors in tandem.

**Additional file 7.** Pyrolysis–GC–MS analysis of wood of transgenic and WT trees.

**Additional file 8.** Expression of lignin and xylan biosynthetic genes.

**Additional file 9.** Monosaccharide composition of non-cellulosic polymers and cellulose content in BMW, LCC fractions, and the residue.

**Additional file 10.** Pyrolysis–GC–MS analysis of BMW, LCC fractions, and the residue.

**Additional file 11.** Primers used for qPCR analysis.

**Additional file 12.** Supplementary methods.

## Abbreviations

BMW: Ball milled wood
DP: Degree of polymerization
FT-IR: Fourier transformed-infrared spectroscopy
G: Guaiacyl
LCC: Lignin–carbohydrate complex
MW: Molecular weight
OLIMP: Oligo mass profiling
PAD: Pulsed amperometric detector
RWA: Reduced wall acetylation
S: Syringyl
WT: Wild type
XOS: Xylo-oligosaccharides
Xyl*p*: Xylopyranosyl
