# Supplementary material for: In muro deacetylation of xylan affects lignin properties and improves saccharification of aspen wood
Source: Biotechnol Biofuels. 2017 Apr 20;10:98. doi: 10.1186/s13068-017-0782-4 (PMC5397736; doi:10.1186/s13068-017-0782-4)
Supplement: Supplementary file 1 — Additional file 1. Transgene expression levels and morphological and mechanical parameters of transgenic trees. [file 13068_2017_782_MOESM1_ESM.pptx]

## Slide 1
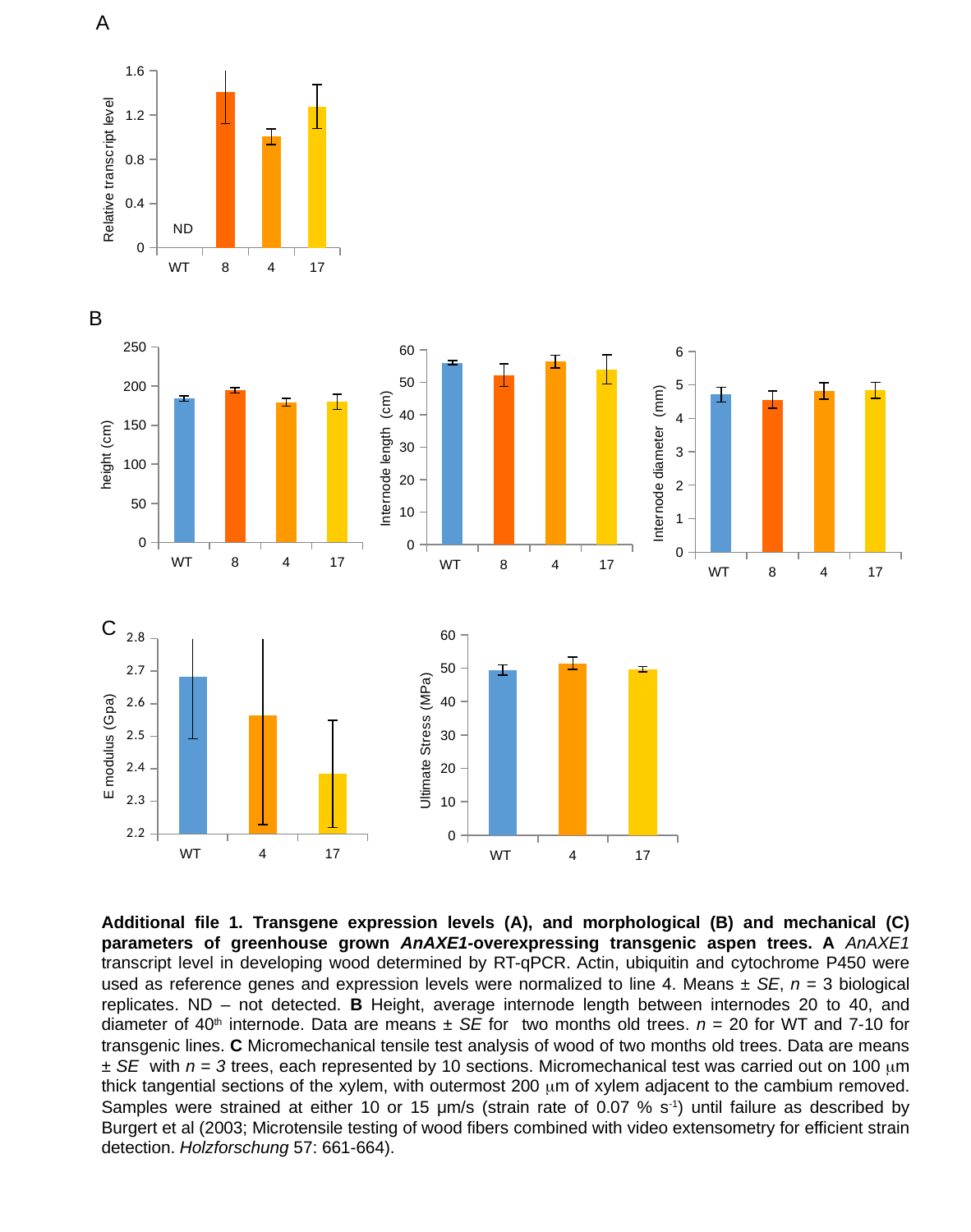

A
### Chart
| Category | |
|---|---|
| WT | 0.0 |
| 8 | 1.406133090341993 |
| 4 | 1.00337177638182 |
| 17 | 1.27424414176091 |B
### Chart
| Category | |
|---|---|
| WT | 184.05 |
| 8 | 194.6666666666666 |
| 4 | 179.4285714285714 |
| 17 | 179.9 |
### Chart
| Category | |
|---|---|
| WT | 56.06666666666643 |
| 8 | 52.22222222222222 |
| 4 | 56.375 |
| 17 | 54.0 |
### Chart
| Category | |
|---|---|
| WT | 4.71 |
| 8 | 4.555555555555535 |
| 4 | 4.819999999999998 |
| 17 | 4.84 |
### Chart
| Category | E-Modul (GPa) |
|---|---|
| WT | 2.684085238095238 |
| 4 | 2.563859956709956 |
| 17 | 2.384613282051282 |
### Chart
| Category | Ultimate Stress (MPa) |
|---|---|
| WT | 49.44485119047619 |
| 4 | 51.46865504329005 |
| 17 | 49.70967847619048 |C
### Chart
| Category |
|---|Additional file 1. Transgene expression levels (A), and morphological (B) and mechanical (C) parameters of greenhouse grown AnAXE1-overexpressing transgenic aspen trees. A AnAXE1 transcript level in developing wood determined by RT-qPCR. Actin, ubiquitin and cytochrome P450 were used as reference genes and expression levels were normalized to line 4. Means ± SE, n = 3 biological replicates. ND – not detected. B Height, average internode length between internodes 20 to 40, and diameter of 40th internode. Data are means ± SE for two months old trees. n = 20 for WT and 7-10 for transgenic lines. C Micromechanical tensile test analysis of wood of two months old trees. Data are means ± SE with n = 3 trees, each represented by 10 sections. Micromechanical test was carried out on 100 mm thick tangential sections of the xylem, with outermost 200 mm of xylem adjacent to the cambium removed. Samples were strained at either 10 or 15 μm/s (strain rate of 0.07 % s-1) until failure as described by Burgert et al (2003; Microtensile testing of wood fibers combined with video extensometry for efficient strain detection. Holzforschung 57: 661-664).
