# Supplementary material for: In muro deacetylation of xylan affects lignin properties and improves saccharification of aspen wood
Source: Biotechnol Biofuels. 2017 Apr 20;10:98. doi: 10.1186/s13068-017-0782-4 (PMC5397736; doi:10.1186/s13068-017-0782-4)
Supplement: Supplementary file 2 — Additional file 2. Transgenic trees have reduced acetylation of xylan. [file 13068_2017_782_MOESM2_ESM.pptx]

## Slide 1
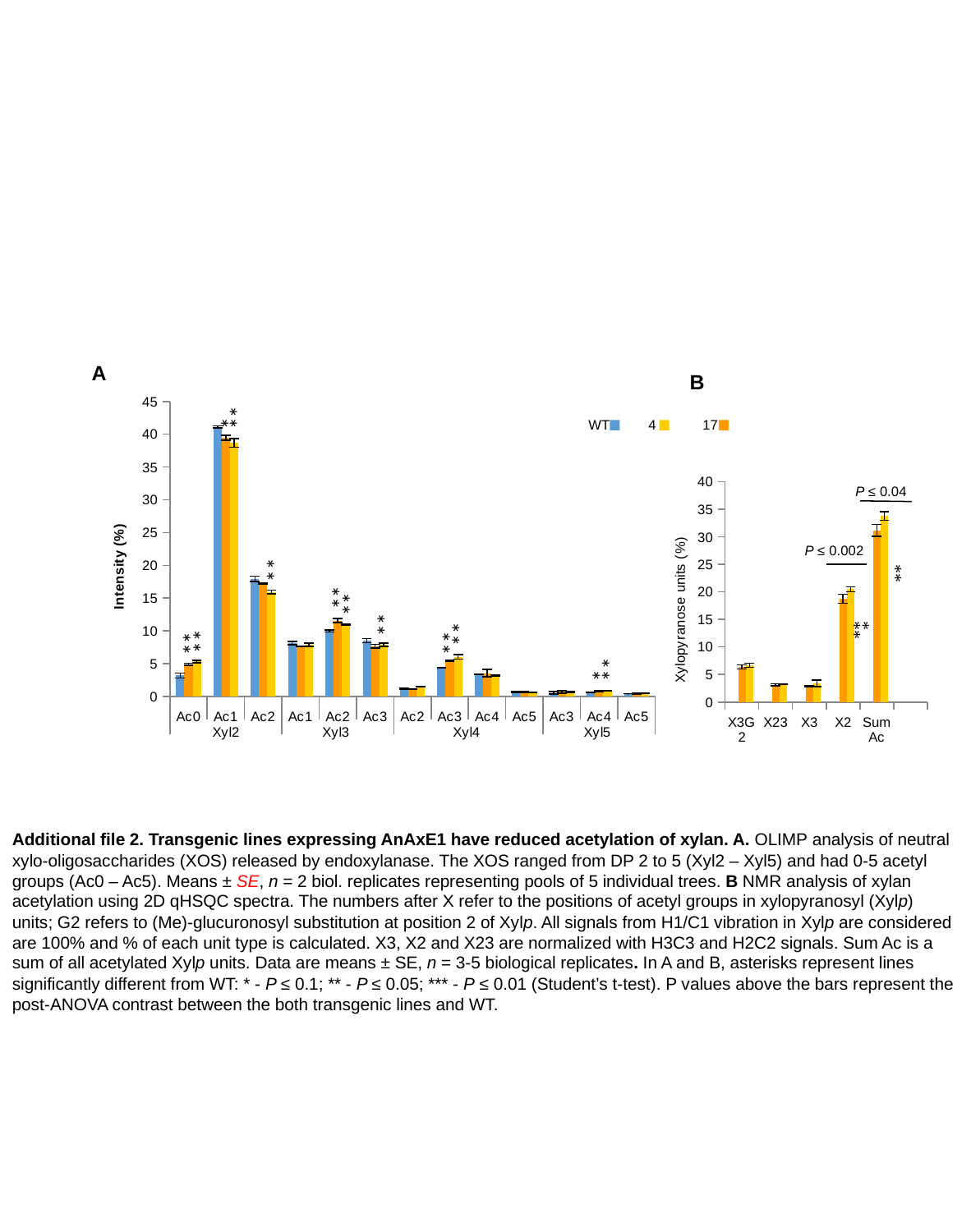

A
B
### Chart
| Category | WT | 4 | 17 |
|---|---|---|---|
| Ac0 | 3.184790180338877 | 4.897628445906534 | 5.313354307365497 |
| Ac1 | 41.14627921778305 | 39.43783177168757 | 38.68403524262932 |
| Ac2 | 17.94532032231371 | 17.24770509796775 | 15.91560607524695 |
| Ac1 | 8.120019630380328 | 7.626636836309267 | 7.874961169903692 |
| Ac2 | 9.97339824437702 | 11.56404654797781 | 10.97118173161443 |
| Ac3 | 8.51444856836788 | 7.657039766506259 | 7.88267994728486 |
| Ac2 | 1.171304116955892 | 1.082030789701484 | 1.457529034970482 |
| Ac3 | 4.394096244017486 | 5.402672776843131 | 6.019737742381743 |
| Ac4 | 3.317207235248832 | 3.546679792599054 | 3.2099696946371 |
| Ac5 | 0.661688354248372 | 0.636952013583842 | 0.636402204477601 |
| Ac3 | 0.538562168549707 | 0.666815814024791 | 0.71190530952814 |
| Ac4 | 0.619174764351608 | 0.804139599342247 | 0.885063002161015 |
| Ac5 | 0.413710953067252 | 0.425453876730813 | 0.437574537799176 |**
*
**
**
**
**
**
**
**
**
*
**
WT 4 17
### Chart
| Category | WT | 4 | 17 |
|---|---|---|---|
| X3G2 | 6.312875606753439 | 6.364949394718645 | 6.66993666363949 |
| X23 | 3.246611127554962 | 3.137971051922061 | 3.26647737881154 |
| X3 | 3.015495833104076 | 2.917537786290223 | 3.382267808880256 |
| X2 | 21.96198238648993 | 18.75471912295404 | 20.4869172875487 |
| Sum Ac | 34.53696495390226 | 31.17517735588497 | 33.80559913888001 |Additional file 2. Transgenic lines expressing AnAxE1 have reduced acetylation of xylan. A. OLIMP analysis of neutral xylo-oligosaccharides (XOS) released by endoxylanase. The XOS ranged from DP 2 to 5 (Xyl2 – Xyl5) and had 0-5 acetyl groups (Ac0 – Ac5). Means ± SE, n = 2 biol. replicates representing pools of 5 individual trees. B NMR analysis of xylan acetylation using 2D qHSQC spectra. The numbers after X refer to the positions of acetyl groups in xylopyranosyl (Xylp) units; G2 refers to (Me)-glucuronosyl substitution at position 2 of Xylp. All signals from H1/C1 vibration in Xylp are considered are 100% and % of each unit type is calculated. X3, X2 and X23 are normalized with H3C3 and H2C2 signals. Sum Ac is a sum of all acetylated Xylp units. Data are means ± SE, n = 3-5 biological replicates. In A and B, asterisks represent lines significantly different from WT: * - P ≤ 0.1; ** - P ≤ 0.05; *** - P ≤ 0.01 (Student’s t-test). P values above the bars represent the post-ANOVA contrast between the both transgenic lines and WT.
