# Supplementary material for: In muro deacetylation of xylan affects lignin properties and improves saccharification of aspen wood
Source: Biotechnol Biofuels. 2017 Apr 20;10:98. doi: 10.1186/s13068-017-0782-4 (PMC5397736; doi:10.1186/s13068-017-0782-4)
Supplement: Supplementary file 3 — Additional file 3. Yield of sugars in pretreatment liquid of transgenic and WT samples. [file 13068_2017_782_MOESM3_ESM.pptx]

## Slide 1
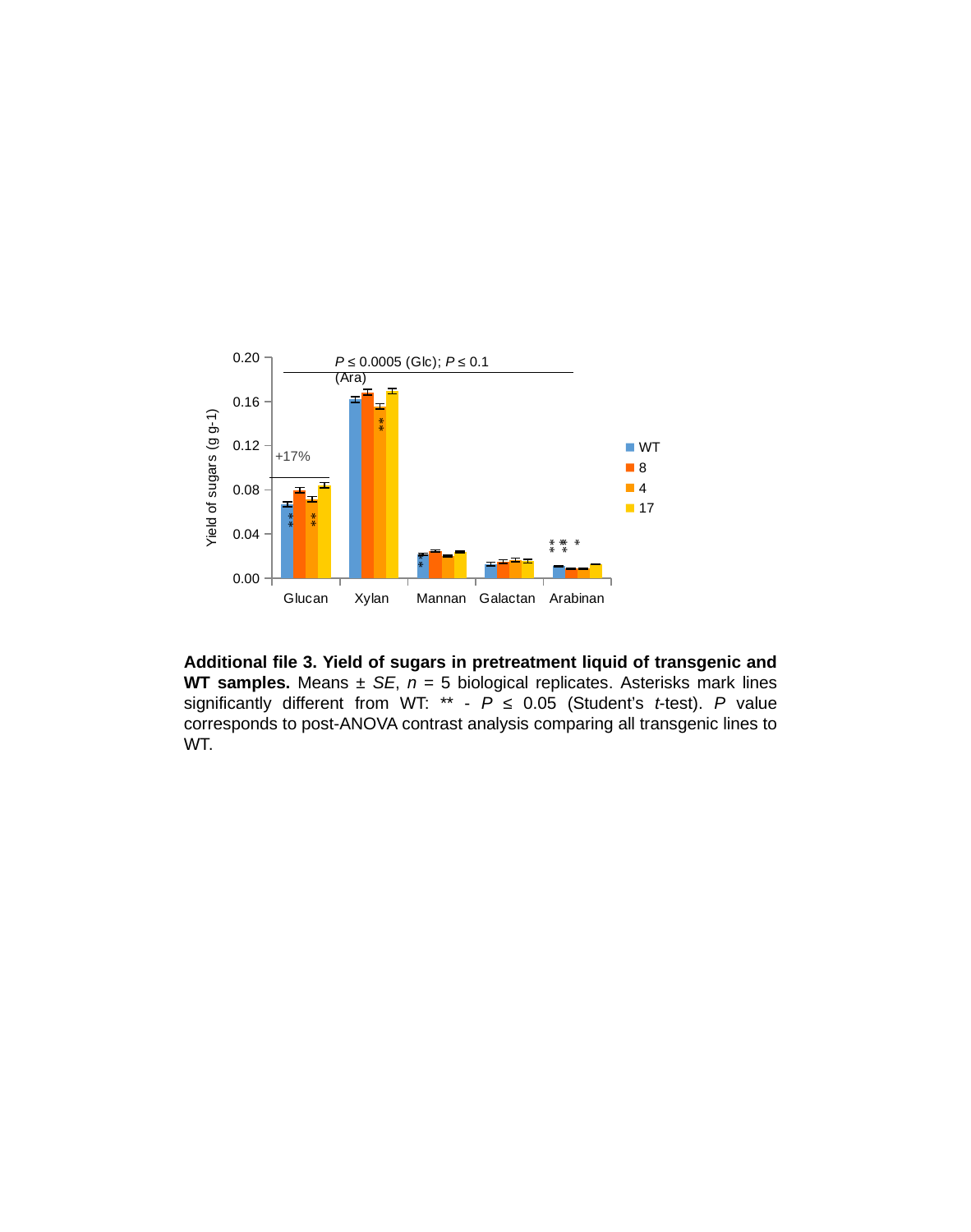

### Chart
| Category | WT | 8 | 4 | 17 |
|---|---|---|---|---|
| Glucan | 0.06684252 | 0.07968763 | 0.07157006 | 0.08412956 |
| Xylan | 0.16179342 | 0.16844658 | 0.15557247 | 0.16949827 |
| Mannan | 0.02175034 | 0.02471229 | 0.01996337 | 0.0236585 |
| Galactan | 0.01275191 | 0.0151471 | 0.0166215 | 0.01547486 |
| Arabinan | 0.01090089 | 0.00862091 | 0.00854504 | 0.01259141 |Additional file 3. Yield of sugars in pretreatment liquid of transgenic and WT samples. Means ± SE, n = 5 biological replicates. Asterisks mark lines significantly different from WT: ** - P ≤ 0.05 (Student’s t-test). P value corresponds to post-ANOVA contrast analysis comparing all transgenic lines to WT.
