# Supplementary material for: In muro deacetylation of xylan affects lignin properties and improves saccharification of aspen wood
Source: Biotechnol Biofuels. 2017 Apr 20;10:98. doi: 10.1186/s13068-017-0782-4 (PMC5397736; doi:10.1186/s13068-017-0782-4)
Supplement: Supplementary file 4 — Additional file 4. Contents of wood components in transgenic trees determined by sequential extractions and weighing. [file 13068_2017_782_MOESM4_ESM.pdf]

Additional file 4. Contents of wood components (mg per g of wood d.w.) in transgenic lines 4 and 17 and wild type (WT) determined by sequential extractions and weighing.

	Extractives	Lignin	Hemicellulose	Cellulose
WT	97.0 ± 3.9	181.0 ± 5.3	249.1 ± 4.3	472.9 ± 0.8
4	99.7 ± 1.2	164.2 ± 7.2	248.0 ± 11.3	488.0 ± 3.5***
17	99.0 ± 07.9	173.6 ± 14.2	254.3 ± 6.3	473.1 ± 6.1

Mean ± SE, n = (3-4) biological replicates. Asterisks next to means of individual lines correspond to means significantly different from wild type (WT) according to the Student´s t-test; *** - $P \leq 0.01$.

Additional file 5. Changes in polysaccharide and lignin composition in *AnAXE1* expressing lines (4, 17) compared to the wild type (WT).

Line	Soluble polysaccharide composition (mol%)								Updegraff cellulose (% wood d.w)	Relative crystallinity (% WT)	
	Ara	Rha	Xyl	Man	MeGlcA	Gal	GalA	Glc		NMR cellulose	XRD sample
WT	1.37 ± 0.05	1.39 ± 0.02	**65.5 ± 0.7****	5.04 ± 0.64	3.11 ± 0.06	**1.53 ± 0.10****	4.98 ± 0.19	**17.1 ± 0.6****	44.6 ± 0.7	**100 ± 0*****	**100 ± 2***
4	1.46 ± 0.03	1.30 ± 0.07	62.3 ± 1.4**	5.66 ± 0.93	2.95 ± 0.14	1.74 ± 0.09	4.68 ± 0.15	19.9 ± 1.3	44.0 ± 0.7	108 ± 2***	102 ± 3
17	1.46 ± 0.06	1.48 ± 0.01	62.9 ± 0.8*	5.48 ± 0.59	3.12 ± 0.08	1.88 ± 0.11	5.25 ± 0.0	18.5 ± 0.2	47.9 ± 2.1	105 ± 4**	106 ± 3*

Mean ± *SE*, *n* = (3-5) biological replicates except for lignin S/G ratio, where *n* = (8-18) biological replicates were analyzed. Asterisks next to individual lines correspond to means significantly different from wild type (WT) according to the post ANOVA Student´s *t*-test. Asterisks next to WT values in bold type denote significance of contrast between WT and all lines. (* - $P \le 0.1$, ** - $P \le 0.5$*, *** - $P \le 0.01$). NMR - nuclear magnetic resonance; XRD = X-ray diffraction.

Additional File 7. Pyrolysis GC-MS analysis of wood of transgenic (lines 8, 4, and 17) and WT trees.

Line	C	G	S	H	P	L	S/G
WT	63.08 ± 0.52	**11.04 ± 0.16****	**18.10 ± 0.24*****	1.89 ± 0.05	0.30 ± 0.01	31.33 ± 0.37	**1.64 ± 0.02*****
8	63.38 ± 0.46	11.41 ± 0.28	17.39 ± 0.35	1.83 ± 0.08	0.28 ± 0.01	30.90 ± 0.65	1.53 ± 0.02**
4	63.51 ± 0.61	12.13 ± 0.19**	16.35 ± 0.4**	1.80 ± 0.09	0.28 ± 0.01	30.56 ± 0.45	1.35 ± 0.04**
17	63.88 ± 0.38	11.09 ± 0.27	17.36 ± 0.19	1.81 ± 0.11	0.27 ± 0.01	30.54 ± 0.40	1.52 ± 0.04*

C-Carbohydrates, G - Guaiacyl lignin, S - Syringyl lignin, H - *p*-hydroxyphenyl, P - phenolics, L - G+S+H+P, S/G - Syringyl to guaiacyl ratio. All values are relative to the sum of detected identified signals. Mean ± SE, n= (8-18) biological replicates. Asteriks next to individual lines correspond to means significantly different from wild type (WT) according to the Student´s t-test. Asterisks next to WT values in bold type denote significance of contrast between WT and transgenic lines (* - $P \leq 0.1$, ** - $P \leq 0.05$, *** - $P \leq 0.01$).

Table S4. Expression of lignin and xylan biosynthetic genes

	F5H	COMT	GT43A	GT43B
WT	1.05 ± 0.22	1.06 ± 0.25	1.04 ± 0.19	1.06 ± 0.23
4	0.71 ± 0.16	0.86 ± 0.16	1.08 ± 0.16	1.33 ± 0.06
8	1.00 ± 0.14	0.84 ± 0.02	0.81 ± 0.13	0.83 ± 0.21
17	1.82 ± 0.72	1.58 ± 0.49	1.3 ± 0.12	1.49 ± 0.35

Transcript level of different genes in developing wood determined by RT-qPCR. Actin, ubiquitin and cytochrome P450 were used as reference genes and expression levels were normalized to WT. Means ± SE, n = 3 biological replicates.

Table S5. Monosaccharide composition of non-cellulosic polymers determined by TMS (mol %) and cellulose content (%; w : w) in ball milled wood (BMW), LCC fractions and the residue in transgenic and WT samples.

Fraction	Line	Ara	Rha	Fuc	Xyl	Man	MeGlcA	Gal	GalA	Glc	GlcA	Updegraff Cellulose
BMW	WT	2.91 ± 0.10	0.78 ± 0.02	0.61 ± 0.00	46.5 ± 1.4	3.69 ± 0.09	4.10 ± 0.11	2.00 ± 0.07	4.20 ± 0.17	34.0 ± 1.6	1.26 ± 0.08	17.7 ± 0.61
	TR	3.13 ± 0.11	0.74 ± 0.04	0.66 ± 0.04	44.1 ± 1.6	3.90 ± 0.10	3.95 ± 0.09	2.12 ± 0.14	4.09 ± 0.13	36.0 ± 1.5	1.35 ± 0.04	16.6 ± 1.68
LCC-X	WT	1.88 ± 0.07	2.57 ± 0.05	0.8 ± 0.09	47.0 ± 1.1	10.65 ± 0.32	2.03 ± 0.09	4.42 ± 0.12	5.86 ± 0.17	23.2 ± 0.3	1.55 ± 0.15	ND
	TR	2.08 ± 0.22	2.59 ± 0.07	0.75 ± 0.21	46.5 ± 0.8	10.47 ± 0.11	2.02 ± 0.18	4.84 ± 0.01*	6.97 ± 0.19*	22.2 ± 0.3*	1.53 ± 0.32	ND
LCC-1	WT	6.05 ± 0.35	5.72 ± 0.09	3.46 ± 0.43	13.5 ± 0.1	6.17 ± 0.18	3.69 ± 0.39	7.67 ± 0.31	42.14 ± 2.84	5.6 ± 0.3	6.00 ± 0.70	ND
	TR	5.61 ± 0.49	5.64 ± 0.16	2.78 ± 0.63	17.7 ± 0.9**	7.01 ± 0.48	3.29 ± 0.56	7.07 ± 0.62	40.34 ± 4.64	5.7 ± 0.4	4.90 ± 1.05	ND
LCC-2	WT	1.86 ± 0.18	1.88 ± 0.05	1.13 ± 0.15	59.6 ± 0.3	3.61 ± 0.03	2.95 ± 0.02	2.20 ± 0.13	5.86 ± 0.23	19.0 ± 0.2	1.93 ± 0.20	17.6 ± 0.97
	TR	2.07 ± 0.17	2.06 ± 0.07	1.25 ± 0.17	58.9 ± 1.9	4.03 ± 0.02**	3.09 ± 0.37	2.54 ± 0.15	6.02 ± 0.17	17.9 ± 1.2	2.10 ± 0.30	17.9 ± 1.75
LCC-3	WT	2.13 ± 0.32	2.28 ± 0.13	1.22 ± 0.36	63.1 ± 2.1	10.94 ± 0.09	3.67 ± 0.15	3.48 ± 0.33	7.14 ± 0.15	3.8 ± 0.1	2.29 ± 0.57	ND
	TR	2.12 ± 0.09	2.34 ± 0.03	1.12 ± 0.09	65.0 ± 0.3	10.25 ± 0.33*	3.78 ± 0.12	3.30 ± 0.07	6.59 ± 0.50	3.3 ± 0.1**	2.17 ± 0.13	ND
Residue	WT	1.49 ± 0.01	1.66 ± 0.03	0.93 ± 0.00	63.4 ± 2.2	2.47 ± 0.05	2.78 ± 0.20	1.86 ± 0.12	4.2 ± 0.14	19.5 ± 1.9	1.73 ± 0.04	31.0 ± 1.01
	TR	1.61 ± 0.08	1.66 ± 0.03	1.03 ± 0.10	60.7 ± 1.4	2.73 ± 0.06**	2.93 ± 0.11	2.02 ± 0.07	3.64 ± 0.10	21.8 ± 0.9**	1.90 ± 0.16	24.2 ± 0.15**

Mean ± SE, n= 3 technical replicates. WT is combination of 6 individual trees. TR is combination of two trees from each transgenic line (8, 4, 17). ND = not detected. Asterisks correspond to means significantly different from WT according to the Fisher´s test (* $P \leq 0.1$, ** $P \leq 0.05$, *** $P \leq 0.01$, ****$P \leq 0.001$).

Table S6. Pyrolysis GC-MS analysis of milled wood powder, different LCC fractions, and the residue.

Fraction	Line	C	G	S	H	P	L	S/G	C/L
milled wood	WT	78.1 ± 0.5	7.72 ± 0.14	11.80 ± 0.08	1.24 ± 0.29	0.08 ± 0.00	20.8 ± 0.4	1.53 ± 0.03	3.75 ± 0.09
	TR	79.1 ± 0.4	7.81 ± 0.21	10.63 ± 0.27	1.41 ± 0.12	0.07 ± 0.01	19.9 ± 0.4	1.36 ± 0.02***	3.97 ± 0.09*
LCC-X	WT	91.1 ± 0.2	2.79 ± 0.05	3.72 ± 0.03	1.34 ± 0.11	0.08 ± 0.01	7.9 ± 0.2	1.33 ± 0.02	11.50 ± 0.26
	TR	91.2 ± 0.1	3.01 ± 0.02***	3.65 ± 0.11	1.24 ± 0.10	0.08 ± 0.01	8.0 ± 0.1	1.21 ± 0.03**	11.42 ± 0.22
LCC-1	WT	79.6 ± 0.7	7.26 ± 0.44	9.06 ± 0.23	2.39 ± 0.05	0.18 ± 0.00	18.9 ± 0.6	1.25 ± 0.04	4.21 ± 0.17
	TR	75.2 ± 0.5***	8.51 ± 0.25**	11.46 ± 0.22***	2.75 ± 0.08**	0.20 ± 0.02	22.9 ± 0.5***	1.35 ± 0.01**	3.29 ± 0.10***
LCC-2	WT	57.5 ± 0.6	11.58 ± 0.17	17.23 ± 0.12	1.96 ± 0.05	0.06 ± 0.01	30.8 ± 0.3	1.49 ± 0.01	1.86 ± 0.03
	TR	59.6 ± 1.0*	11.92 ± 0.73	15.85 ± 0.78*	2.05 ± 0.30	0.08 ± 0.00	29.9 ± 1.3	1.33 ± 0.04**	2.00 ± 0.09
LCC-3	WT	51.1 ± 0.4	14.36 ± 0.49	23.37 ± 0.35	2.83 ± 0.14	0.25 ± 0.01	40.8 ± 0.7	1.63 ± 0.03	1.25 ± 0.01
	TR	53.1 ± 0.5	14.05 ± 0.53	21.63 ± 1.04	2.69 ± 0.10	0.25 ± 0.00	38.6 ± 1.5	1.55 ± 0.02**	1.39 ± 0.06
Residue	WT	50.2 ± 1.8	6.27 ± 0.71	10.84 ± 1.09	0.82 ± 0.06	0.04 ± 0.00	18.0 ± 1.7	1.73 ± 0.02	2.86 ± 0.13
	TR	57.9 ± 1.0	6.46 ± 0.43	9.54 ± 0.65	0.63 ± 0.01**	0.03 ± 0.00	16.7 ± 1.1	1.48 ± 0.00**	3.48 ± 0.16**

C-Carbohydrates, G - Guaiacyl lignin, S - Syringyl lignin, H - *p*-hydroxyphenyl, P - phenolics, L - G+S+H+P, S/G - Syringyl to Guaiacyl ratio. All values are relative. Mean ± SE, n= 3 technical replicates. WT is combination of 6 individual trees. Trans is combination of two trees from each transgenic line (8, 4, 17). Asterisks correspond to means significantly different from WT

Table S7. Primers used for qPCR analysis

Oligo name	Target gene ID	Name of gene	Length of oligos	Sequence (5´-3´)	Efficiency
DFC2_1F	CAA01634	AXE	194	CTGGACGATTAGCGAGTACG	2.05
DFC2_1R				CGCAGGTGGAATTCCATCC	
ACT11 for	Potri.006G192700	ACTIN	208	TATTGTTCTCAGTGGTGGCTCT	1.9
ACT11 rev				GGACTCATCATACTCTGCCTTT	
CYP for	Potri.004G168800	CYP	234	TAAGACCGAATGGCTTGACG	2
CYP rev				AGAACGCACCCCAAAACTACTA	
TUB_for	Potri.001G464400	TUB	200	ATTCCCTCGCCTTCATTTCT	1.9
TUB_rev				CCTCTTTCGTGCTCATCTTACC	
UBQ-L_for	Potri.005G198700	UBQ	200	TGGCAAGACCATAACTCTCG	1.86
UBQ-L_rev				CTCCCCTAAGCCTCAAAACC	
COMT-F	Potri.012G006400	COMT	125	AGCACAATCGTCTCCAAGTACCCT	2.02
COMT-R				AACATTCTCCACACCAGGGAAAGC	
F5H-F	Potri.007G016400	F5H	125	AAGCCAATATAGGCAAGCCTGTGAATC	1.86
F5H-R				ATTTTTAGCCCCGAAAGCTGCTCTG	
GT43Afor1	Potri.006G131000	GT43A	213	GTCGCCCTTCATCTGTCC	2.11
GT43Arev1				TCCCTCATAGTTTTCTCCTGCT	
GT43Bfor1	Potri.016G086400	GT43B	183	GTCGCCCTTCTTCAGTCCAG	2.01
GT43Brev1				TTTTGTCTTCTTGATTTTCCTGA	
