# Supplementary material for: In muro deacetylation of xylan affects lignin properties and improves saccharification of aspen wood
Source: Biotechnol Biofuels. 2017 Apr 20;10:98. doi: 10.1186/s13068-017-0782-4 (PMC5397736; doi:10.1186/s13068-017-0782-4)
Supplement: Supplementary file 5 — Additional file 5. Changes in polysaccharide composition and crystallinity in transgenic trees. [file 13068_2017_782_MOESM5_ESM.pdf]

Additional file 5. Changes in polysaccharide composition and cellulose in *AnAXE1* expressing lines (4, 17) compared to the wild type (WT).

**Line**	**Soluble polysaccharide composition (mol%)**								**Updegraff cellulose (% wood d.w)**	**Relative crystallinity (% WT)**	
	**Ara**	**Rha**	**Xyl**	**Man**	**MeGlcA**	**Gal**	**GalA**	**Glc**		**NMR cellulose**	**XRD sample**
WT	1.37 ± 0.05	1.39 ± 0.02	**65.5 ± 0.7****	5.04 ± 0.64	3.11 ± 0.06	**1.53 ± 0.10****	4.98 ± 0.19	**17.1 ± 0.6****	44.6 ± 0.7	**100 ± 0*****	**100 ± 2***
4	1.46 ± 0.03	1.30 ± 0.07	62.3 ± 1.4**	5.66 ± 0.93	2.95 ± 0.14	1.74 ± 0.09	4.68 ± 0.15	19.9 ± 1.3	44.0 ± 0.7	108 ± 2***	102 ± 3
17	1.46 ± 0.06	1.48 ± 0.01	62.9 ± 0.8*	5.48 ± 0.59	3.12 ± 0.08	1.88 ± 0.11	5.25 ± 0.0	18.5 ± 0.2	47.9 ± 2.1	105 ± 4**	106 ± 3*

Mean ± *SE*, *n* = (3-5) biological replicates except for lignin S/G ratio, where *n* = (8-18) biological replicates were analyzed. Asterisks next to individual lines correspond to means significantly different from wild type (WT) according to the post ANOVA Student´s *t*-test. Asterisks next to WT values in bold type denote significance of contrast between WT and all lines. (* - $P \leq 0.1$, ** - $P \leq 0.5$*, *** - $P \leq 0.01$). NMR - nuclear magnetic resonance; XRD = X-ray diffraction.
