# Supplementary material for: In muro deacetylation of xylan affects lignin properties and improves saccharification of aspen wood
Source: Biotechnol Biofuels. 2017 Apr 20;10:98. doi: 10.1186/s13068-017-0782-4 (PMC5397736; doi:10.1186/s13068-017-0782-4)
Supplement: Supplementary file 6 — Additional file 6. Size exclusion chromatography of xylan extracted from wood using UV and PAD detectors in tandem. [file 13068_2017_782_MOESM6_ESM.pptx]

## Slide 1
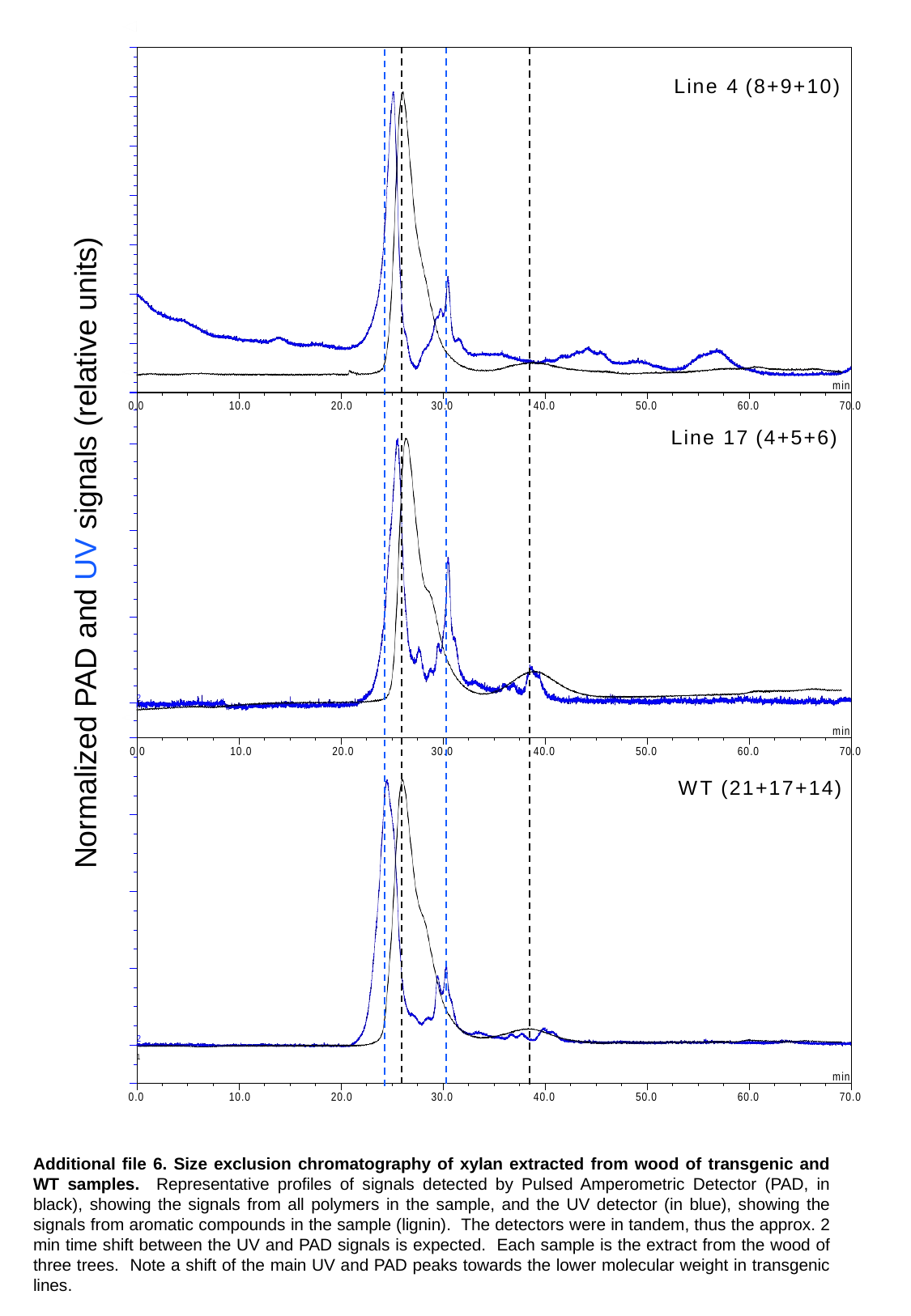

UV_VIS_1
min
0.0	10.0	20.0
30.0
40.0
50.0
60.0
70.0
Line 4 (8+9+10)
1186_05
UV_VIS_1
Line 17 (4+5+6)
2
1
min
0.0	10.0	20.0
30.0
40.0
50.0
60.0
70.0
Normalized PAD and UV signals (relative units)
1186_08
 WT (21+17+14)
21
min
0.0	10.0	20.0
30.0
40.0
50.0
60.0
70.0
Additional file 6. Size exclusion chromatography of xylan extracted from wood of transgenic and WT samples. Representative profiles of signals detected by Pulsed Amperometric Detector (PAD, in black), showing the signals from all polymers in the sample, and the UV detector (in blue), showing the signals from aromatic compounds in the sample (lignin). The detectors were in tandem, thus the approx. 2 min time shift between the UV and PAD signals is expected. Each sample is the extract from the wood of three trees. Note a shift of the main UV and PAD peaks towards the lower molecular weight in transgenic lines.
