# Supplementary material for: In muro deacetylation of xylan affects lignin properties and improves saccharification of aspen wood
Source: Biotechnol Biofuels. 2017 Apr 20;10:98. doi: 10.1186/s13068-017-0782-4 (PMC5397736; doi:10.1186/s13068-017-0782-4)
Supplement: Supplementary file 7 — Additional file 7. Pyrolysis–GC–MS analysis of wood of transgenic and WT trees. [file 13068_2017_782_MOESM7_ESM.pdf]

Additional file 7. Pyrolysis GC-MS analysis of wood of transgenic (lines 8, 4, and 17) and wild type (WT) trees.

Line	C	G	S	H	P	L	S/G
WT	63.08 ± 0.52	**11.04 ± 0.16****	**18.10 ± 0.24*****	1.89 ± 0.05	0.30 ± 0.01	31.33 ± 0.37	**1.64 ± 0.02*****
8	63.38 ± 0.46	11.41 ± 0.28	17.39 ± 0.35	1.83 ± 0.08	0.28 ± 0.01	30.90 ± 0.65	1.53 ± 0.02**
4	63.51 ± 0.61	12.13 ± 0.19**	16.35 ± 0.4**	1.80 ± 0.09	0.28 ± 0.01	30.56 ± 0.45	1.35 ± 0.04**
17	63.88 ± 0.38	11.09 ± 0.27	17.36 ± 0.19	1.81 ± 0.11	0.27 ± 0.01	30.54 ± 0.40	1.52 ± 0.04*

C-Carbohydrates, G - Guaiacyl lignin, S - Syringyl lignin, H - *p*-hydroxyphenyl, P - phenolics, L - G+S+H+P, S/G - Syringyl to guaiacyl ratio. All values are relative to the sum of detected identified signals. Mean ± *SE*, $n =$ (8-18) biological replicates. Asteriks next to individual lines correspond to means significantly different from wild type (WT) according to the Student´s t-test. Asterisks next to WT values in bold type denote significance of contrast between WT and transgenic lines (* - $P \leq 0.1$, ** - $P \leq 0.05$, *** - $P \leq 0.01$).
