# Supplementary material for: In muro deacetylation of xylan affects lignin properties and improves saccharification of aspen wood
Source: Biotechnol Biofuels. 2017 Apr 20;10:98. doi: 10.1186/s13068-017-0782-4 (PMC5397736; doi:10.1186/s13068-017-0782-4)
Supplement: Supplementary file 8 — Additional file 8. Expression of lignin and xylan biosynthetic genes. [file 13068_2017_782_MOESM8_ESM.pdf]

Additional file 8. Expression of lignin and xylan biosynthetic genes

	F5H	*COMT*	*GT43A*	*GT43B*
WT	1.05 ± 0.22	1.06 ± 0.25	1.04 ± 0.19	1.06 ± 0.23
4	0.71 ± 0.16	0.86 ± 0.16	1.08 ± 0.16	1.33 ± 0.06
8	1.00 ± 0.14	0.84 ± 0.02	0.81 ± 0.13	0.83 ± 0.21
17	1.82 ± 0.72	1.58 ± 0.49	1.3 ± 0.12	1.49 ± 0.35

Transcript level of different genes in developing wood determined by RT-qPCR. Actin, ubiquitin and cytochrome P450 were used as reference genes and expression levels were normalized to WT. Means ± SE, $n = 3$ biological replicates.
