# Supplementary material for: In muro deacetylation of xylan affects lignin properties and improves saccharification of aspen wood
Source: Biotechnol Biofuels. 2017 Apr 20;10:98. doi: 10.1186/s13068-017-0782-4 (PMC5397736; doi:10.1186/s13068-017-0782-4)
Supplement: Supplementary file 9 — Additional file 9. Monosaccharide composition of non-cellulosic polymers and cellulose content in BMW, LCC fractions, and the residue. [file 13068_2017_782_MOESM9_ESM.pdf]

Additional file 9. Monosaccharide composition of non-cellulosic polymers determined by TMS (mol %) and cellulose content (%; w : w) in ball milled wood (BMW), LCC fractions and the residue in transgenic and wild type (WT) samples.

Fraction	Line	Ara	Rha	Fuc	Xyl	Man	MeGlcA	Gal	GalA	Glc	GlcA	Updegraff Cellulose
BMW	WT	2.91 ± 0.10	0.78 ± 0.02	0.61 ± 0.00	46.5 ± 1.4	3.69 ± 0.09	4.10 ± 0.11	2.00 ± 0.07	4.20 ± 0.17	34.0 ± 1.6	1.26 ± 0.08	17.7 ± 0.61
	TR	3.13 ± 0.11	0.74 ± 0.04	0.66 ± 0.04	44.1 ± 1.6	3.90 ± 0.10	3.95 ± 0.09	2.12 ± 0.14	4.09 ± 0.13	36.0 ± 1.5	1.35 ± 0.04	16.6 ± 1.68
LCC-X	WT	1.88 ± 0.07	2.57 ± 0.05	0.8 ± 0.09	47.0 ± 1.1	10.65 ± 0.32	2.03 ± 0.09	4.42 ± 0.12	5.86 ± 0.17	23.2 ± 0.3	1.55 ± 0.15	ND
	TR	2.08 ± 0.22	2.59 ± 0.07	0.75 ± 0.21	46.5 ± 0.8	10.47 ± 0.11	2.02 ± 0.18	4.84 ± 0.01*	6.97 ± 0.19*	22.2 ± 0.3*	1.53 ± 0.32	ND
LCC-1	WT	6.05 ± 0.35	5.72 ± 0.09	3.46 ± 0.43	13.5 ± 0.1	6.17 ± 0.18	3.69 ± 0.39	7.67 ± 0.31	42.14 ± 2.84	5.6 ± 0.3	6.00 ± 0.70	ND
	TR	5.61 ± 0.49	5.64 ± 0.16	2.78 ± 0.63	17.7 ± 0.9**	7.01 ± 0.48	3.29 ± 0.56	7.07 ± 0.62	40.34 ± 4.64	5.7 ± 0.4	4.90 ± 1.05	ND
LCC-2	WT	1.86 ± 0.18	1.88 ± 0.05	1.13 ± 0.15	59.6 ± 0.3	3.61 ± 0.03	2.95 ± 0.02	2.20 ± 0.13	5.86 ± 0.23	19.0 ± 0.2	1.93 ± 0.20	17.6 ± 0.97
	TR	2.07 ± 0.17	2.06 ± 0.07	1.25 ± 0.17	58.9 ± 1.9	4.03 ± 0.02**	3.09 ± 0.37	2.54 ± 0.15	6.02 ± 0.17	17.9 ± 1.2	2.10 ± 0.30	17.9 ± 1.75
LCC-3	WT	2.13 ± 0.32	2.28 ± 0.13	1.22 ± 0.36	63.1 ± 2.1	10.94 ± 0.09	3.67 ± 0.15	3.48 ± 0.33	7.14 ± 0.15	3.8 ± 0.1	2.29 ± 0.57	ND
	TR	2.12 ± 0.09	2.34 ± 0.03	1.12 ± 0.09	65.0 ± 0.3	10.25 ± 0.33*	3.78 ± 0.12	3.30 ± 0.07	6.59 ± 0.50	3.3 ± 0.1**	2.17 ± 0.13	ND
Residue	WT	1.49 ± 0.01	1.66 ± 0.03	0.93 ± 0.00	63.4 ± 2.2	2.47 ± 0.05	2.78 ± 0.20	1.86 ± 0.12	4.2 ± 0.14	19.5 ± 1.9	1.73 ± 0.04	31.0 ± 1.01
	TR	1.61 ± 0.08	1.66 ± 0.03	1.03 ± 0.10	60.7 ± 1.4	2.73 ± 0.06**	2.93 ± 0.11	2.02 ± 0.07	3.64 ± 0.10	21.8 ± 0.9**	1.90 ± 0.16	24.2 ± 0.15**

Mean ± SE, n = 3 technical replicates. WT is combination of 6 individual trees. TR is combination of two trees from each transgenic line (8, 4, 17). ND = not detected. Asterisks correspond to means significantly different from WT according to the ANOVA ($*P \le 0.1$, $**P \le 0.05$, $***P \le 0.01$, $****P \le 0.001$).
