# Supplementary material for: In muro deacetylation of xylan affects lignin properties and improves saccharification of aspen wood
Source: Biotechnol Biofuels. 2017 Apr 20;10:98. doi: 10.1186/s13068-017-0782-4 (PMC5397736; doi:10.1186/s13068-017-0782-4)
Supplement: Supplementary file 10 — Additional file 10. Pyrolysis–GC–MS analysis of BMW, LCC fractions, and the residue. [file 13068_2017_782_MOESM10_ESM.pdf]

Additional file 10. Pyrolysis GC-MS analysis of ball milled wood (BMW), different LCC fractions, and the residue.

Fraction	Line	C	G	S	H	P	L	S/G	C/L
BMW	WT	78.1 ± 0.5	7.72 ± 0.14	11.80 ± 0.08	1.24 ± 0.29	0.08 ± 0.00	20.8 ± 0.4	1.53 ± 0.03	3.75 ± 0.09
	TR	79.1 ± 0.4	7.81 ± 0.21	10.63 ± 0.27	1.41 ± 0.12	0.07 ± 0.01	19.9 ± 0.4	1.36 ± 0.02***	3.97 ± 0.09*
LCC-X	WT	91.1 ± 0.2	2.79 ± 0.05	3.72 ± 0.03	1.34 ± 0.11	0.08 ± 0.01	7.9 ± 0.2	1.33 ± 0.02	11.50 ± 0.26
	TR	91.2 ± 0.1	3.01 ± 0.02***	3.65 ± 0.11	1.24 ± 0.10	0.08 ± 0.01	8.0 ± 0.1	1.21 ± 0.03**	11.42 ± 0.22
LCC-1	WT	79.6 ± 0.7	7.26 ± 0.44	9.06 ± 0.23	2.39 ± 0.05	0.18 ± 0.00	18.9 ± 0.6	1.25 ± 0.04	4.21 ± 0.17
	TR	75.2 ± 0.5***	8.51 ± 0.25**	11.46 ± 0.22***	2.75 ± 0.08**	0.20 ± 0.02	22.9 ± 0.5***	1.35 ± 0.01**	3.29 ± 0.10***
LCC-2	WT	57.5 ± 0.6	11.58 ± 0.17	17.23 ± 0.12	1.96 ± 0.05	0.06 ± 0.01	30.8 ± 0.3	1.49 ± 0.01	1.86 ± 0.03
	TR	59.6 ± 1.0*	11.92 ± 0.73	15.85 ± 0.78*	2.05 ± 0.30	0.08 ± 0.00	29.9 ± 1.3	1.33 ± 0.04**	2.00 ± 0.09
LCC-3	WT	51.1 ± 0.4	14.36 ± 0.49	23.37 ± 0.35	2.83 ± 0.14	0.25 ± 0.01	40.8 ± 0.7	1.63 ± 0.03	1.25 ± 0.01
	TR	53.1 ± 0.5	14.05 ± 0.53	21.63 ± 1.04	2.69 ± 0.10	0.25 ± 0.00	38.6 ± 1.5	1.55 ± 0.02**	1.39 ± 0.06
Residue	WT	50.2 ± 1.8	6.27 ± 0.71	10.84 ± 1.09	0.82 ± 0.06	0.04 ± 0.00	18.0 ± 1.7	1.73 ± 0.02	2.86 ± 0.13
	TR	57.9 ± 1.0	6.46 ± 0.43	9.54 ± 0.65	0.63 ± 0.01**	0.03 ± 0.00	16.7 ± 1.1	1.48 ± 0.00**	3.48 ± 0.16**

C-Carbohydrates, G - Guaiacyl lignin, S - Syringyl lignin, H - *p*-hydroxyphenyl, P - phenolics, L - G+S+H+P, S/G - Syringyl to Guaiacyl ratio. All values are relative. Mean ± *SE*, $n = 3$ technical replicates. WT is combination of 6 individual trees. Trans is combination of two trees from each transgenic line (8, 4, 17). Asterisks correspond to means significantly different from WT according to the ANOVA (* $P \leq 0.1$, ** $P \leq 0.05$, *** $P \leq 0.01$).
