# Supplementary material for: In muro deacetylation of xylan affects lignin properties and improves saccharification of aspen wood
Source: Biotechnol Biofuels. 2017 Apr 20;10:98. doi: 10.1186/s13068-017-0782-4 (PMC5397736; doi:10.1186/s13068-017-0782-4)
Supplement: Supplementary file 11 — Additional file 11. Primers used for qPCR analysis. [file 13068_2017_782_MOESM11_ESM.pdf]

Additional file 11. Primers used for qPCR analysis

Oligo name	Target gene ID	Name of gene	Length of oligos	Sequence (5´-3´)	Efficiency
DFC2_1F	CAA01634	AXE	194	CTGGACGATTAGCGAGTACG	2.1
DFC2_1R				CGCAGGTGGAATTCCATCC	
ACT11 for	Potri.006G192700	ACTIN	208	TATTGTTCTCAGTGGTGGCTCT	1.9
ACT11 rev				GGACTCATCATACTCTGCCTTT	
CYP for	Potri.004G168800	CYP	234	TAAGACCGAATGGCTTGACG	2.0
CYP rev				AGAACGCACCCCAAAACTACTA	
TUB_for	Potri.001G464400	TUB	200	ATTCCCTCGCCTTCATTTCT	1.9
TUB_rev				CCTCTTTCGTGCTCATCTTACC	
UBQ-L_for	Potri.005G198700	UBQ	200	TGGCAAGACCATAACTCTCG	1.9
UBQ-L_rev				CTCCCCTAAGCCTCAAAACC	
COMT-F	Potri.012G006400	COMT	125	AGCACAATCGTCTCCAAGTACCCT	2.0
COMT-R				AACATTCTCCACACCAGGGAAAGC	
F5H-F	Potri.007G016400	F5H	125	AAGCCAATATAGGCAAGCCTGTGAATC	1.9
F5H-R				ATTTTTAGCCCCGAAAGCTGCTCTG	
GT43Afor1	Potri.006G131000	GT43A	213	GTCGCCCTTCATCTGTCC	2.1
GT43Arev1				TCCCTCATAGTTTTCTCCTGCT	
GT43Bfor1	Potri.016G086400	GT43B	183	GTCGCCCTTCTTCAGTCCAG	2.0
GT43Brev1				TTTTGTCTTCTTGATTTTCCTGA	
