# Supplementary material for: In muro deacetylation of xylan affects lignin properties and improves saccharification of aspen wood
Source: Biotechnol Biofuels. 2017 Apr 20;10:98. doi: 10.1186/s13068-017-0782-4 (PMC5397736; doi:10.1186/s13068-017-0782-4)
Supplement: Supplementary file 12 — Additional file 12. Supplementary methods. [file 13068_2017_782_MOESM12_ESM.docx]

**Supplemental Methods**

**Crystallinity of cellulose by Nuclear Magnetic Resonance (NMR)**

To 100 mg of AIR, 40 μl of de-ionized water was added, mixed and packed into 4 mm MAS rotors. Spectra were recorded on a 500 MHz Bruker DRX spectrometer operating at 125.75 MHz, equipped with a 4 mm MAS probe. Spin-rate was set to 7000 Hz and 5120 scans were collected for each sample. A Gaussian apodization was used before Fourier transform. Line-shape analysis using Gaussian shapes was used to approximate the integrals for the crystalline (89 ppm) and amorphous (84 ppm) C-4 regions. Topspin 3.1 (Bruker Biospin, Rheinstetten, Germany) was used for processing and analysis.

**Sample crystallinity by X-ray diffraction**

AIR was measured using perpendicular transmission geometry using the two-dimensional scattering set-up and the data corrections presented in (1). Samples were measured in metal rings between thin Mylar foils. A linear combination of Gaussian peaks representing cellulose Iβ (2) reflections and an amorphous model represented by a sulphate-lignin scattering data (3) were fitted to the corrected scattering intensities. Sample crystallinity was calculated as the mass fraction of crystalline cellulose in the sample (with scattering angles of 13 to 50°) and the values were normalized with the corresponding WT values that were set to 100%.

1. Leppanen K, et al. (2011) X-ray scattering and microtomography study on the structural changes of never-dried silver birch, European aspen and hybrid aspen during drying. *Holzforschung* 65:865-873.

2. Nishiyama Y, Langan P, & Chanzy H (2002) Crystal structure and hydrogen-bonding system in cellulose 1 beta from synchrotron X-ray and neutron fiber diffraction. *J Am Chem Soc* 124:9074-9082.

3. Andersson S, Serimaa R, Paakkari T, Saranpaa P, & Pesonen E (2003) Crystallinity of wood and the size of cellulose crystallites in Norway spruce (*Picea abies*). *J Wood Sci* 49:531-537.

**Xylan analysis by OLIMP**

Alcohol insoluble residue (AIR) was prepared from fine wood powder by sequential treatments with 80% ethanol (made with 4 mM, HEPES buffer, pH 7.5), methanol : chloroform (1:1, v/v) and acetone, and vacuum drying. AIR was heat-treated at 100 ˚C for 5 min to deactivate enzymes, digested using GH10 endo-1,4-β-D xylanase from *Aspergillus aculeatus* (gift from Novozymes), desalted and separated using Hypersep Hypercarb Porous Graphitized Carbon (PGC) columns (Thermo Scientific, Waltham, MA, USA) into acidic and neutral fractions, which were freeze dried and dissolved in water for AP-MALDI-ITMS analysis (4).

4. Chong SL, et al. (2011) Feasibility of using atmospheric pressure matrix-assisted laser desorption/ionization with ion trap mass spectrometry in the analysis of acetylated xylooligosaccharides derived from hardwoods and *Arabidopsis thaliana*. *Anal Bioanal Chem* 401:2995-3009.

**Xylan analysis by NMR**

300 mg of alcohol insoluble residue was delignified using peracetic acid solution (32% in 40-45% acetic acid, Sigma-Aldrich, Sweden) at 85^◦^C for 20 min, and extracted twice with DMSO at 60^◦^C for 24 h. Combined supernatants were precipitated using five volumes of ethanol:methanol:water (7:2:1, pH 7.3 adjusted with formic acid) at 4^◦^C for 3 days. The precipitate was mixed with 20 μl of water, freeze dried, and the NMR spectra of the samples rehydrated with 500 µL D_2_O were acquired using a Bruker Avance III HD 850 MHz spectrometer equipped with a TCI HCN cryoprobe operating at 213.78 MHz 13C frequency. A Bruker SampleXpress sample changer and ICON-NMR software (Bruker Biospin, Rheinstetten, Germany) were used to record spectra. The 2D 1H-13C HSQC spectra with multiplicity editing (hsqcedetgpsisp2.3) were obtained at 298 K, with 320 increments, 12 transients, 2 s relaxation delay, sweep widths of 7 and 120 ppm, and optimized for a direct coupling constant of 145 Hz. The total acquisition time was 142 min. Topspin 3.2 (Bruker Biospin) was used for processing and analysis.

**Xylan size exclusion chromatography**

300 mg of extractives-free fine wood powder was boiled in 10 ml of 1% ammonium oxalate (pH 5) for 2 h, and the pellet was treated with 10 ml of 4 M KOH with 3% H_3_BO_3_ for 24 h with continuous rotation. 10 ml of supernatant was precipitated with 30 ml of ethanol-glacial acetic acid mixture (7:3, v:v) at 4^◦^C overnight, freeze dried, dissolved in water, filtered through 45 μm mesh, and analyzed by size exclusion chromatography with pulsed amperometric detector in tandem with a UV detector at MoRe Research (Örnsköldsvik, Sweden). The calibration was done with pullulan standards with known molecular weights.

**Pretreatment and saccharification of wood**

Rough wood powder was sieved to the particle size 0.1 - 0.5 mm using an analytical sieve shaker (Retsch AS 200) and combined from two trees as one biological replicate using equal weights. 50 mg of sample was pretreated with 1% sulphuric acid (w/w) using a single-mode microwave system (Initiator Exp, Biotage, Uppsala, Sweden) at 165 °C for 10 min. The pretreatment liquid and the residue were collected. The residue was washed twice with deionized water and once with sodium citrate buffer (50 mM, pH 5.2), and incubated at 45 °C for 72 h with 50 mg of a 1:1 (w/w) mixture of Celluclast 1.5 L (a cellulase-rich liquid enzyme preparation from *Trichoderma reesei* ATCC 26921 with a stated activity of 700 endoglucanase units per g) and Novozyme 188 (a cellobiase-rich liquid enzyme preparation from *A. niger* with a stated activity of 250 cellobiase units per g (both from Sigma Aldrich). The sodium citrate buffer was added to a total reaction mixture weight of 1000 mg. The same procedure was used for enzymatic hydrolysis of 50 mg of wood powder without pretreatment. The liquid fractions were analyzed using high-performance anion-exchange chromatography (HPAEC), as previously described (5).

5. Gandla ML, et al. (2015) Expression of a fungal glucuronoyl esterase in *Populus*: Effects on wood properties and saccharification efficiency. *Phytochemistry* 112:210-220.

**Preparation of LCC fractions**

The LCC fractionation procedure was modified from (5). To inactivate enzymes, 11.6 g of BMW was treated with 300 mL water at 60 ^◦^C for 3 h. The pellet was separated by centrifugation at 5200 *g* for 10 min. This centrifugation setting was followed in the subsequent steps. The supernatant was concentrated and freeze-dried, and called LCC-X. The pellet was digested with 35 mL of Fibercare containing a mixture of endoglucanases (gift from Novozymes) and 350 mL of acetate buffer (0.05 M, pH 5.6) for 38 h in a shaker set at 150 rpm at 50 ^◦^C. The supernatant was separated from the pellet by centrifugation and evaporated with low pressure at 50 ^◦^C to half of its initial volume. 10 mL of saturated Ba(OH)_2_ was added to this solution and incubated overnight at room temperature with gentle stirring. The resulting precipitate was collected by centrifugation, dissolved in 10 mL of glacial acetic acid, reprecipitated in 40 mL of absolute ethanol, collected by centrifugation, dissolved in a small amount of water, dialyzed in a Cellulose RC dialysis membrane (1000 MWCO, Spectrum Labs, Rancho Dominguez, USA) against water, and freeze-dried (LCC-1). The pellet after endoglucanase digestion was treated with 200 mL of DMSO at 60 ^◦^C overnight. The supernatant was collected after centrifugation and the pellet was treated with DMSO again. Two supernatants were combined and saved, and the residue was washed twice with water and freeze-dried (Residue). The combined supernatants (400 mL) were mixed with sodium chloride at a final concentration of 1.25% (w/v), stirred for 2 h, and centrifuged. The pellet was dissolved in water, followed by dialysis and freeze-drying to yield LCC-2 fraction. The supernatant was precipitated with four volumes of ethanol overnight at 4 ^◦^C. The precipitate was collected by centrifugation and dissolved in a small amount of water. This solution was dialyzed against water, freeze-dried, and labelled as fraction LCC-3.

*Dioxane-water soluble lignin:* 10 mg of BMW was treated with 10 mL of a dioxane:water mixture (96:4; v:v) and stirred at room temperature for 48 h. The supernatant was separated by centrifugation and the absorbance was measured at 280 nm. The concentration of lignin was calculated assuming an absorptivity of 18.21 [Lg^-1^cm^-1^] (40).^.^

*Acidic dioxane soluble lignin:* 2 g of BMW was incubated in 80 mL of a dioxane:water mixture (96:4; v/v) supplemented with 0.66 mL 12 M HCl for 2 h at 100°C. The solution was cooled, filtered, and the residue was washed with a dioxane:water (96:4) mixture. 150 mL of water was added to the combined filtrates and the volume was decreased to 50 ml by rotary evaporation. 450 mL of water was added to this solution, which was incubated O/N for precipitation. The supernatant was slowly removed and the residue was washed with a 0.01 M aqueous solution of HCl followed by centrifugation at 5200 *g* for 20 min. The pellet was mixed with 5 mL of water and freeze dried. It was used for determination of acetyl bromide soluble lignin, TMS sugars, GC-MS pyrolysis, and Updegraff cellulose, as described above.

5. Lawoko M (2013) Unveiling the structure and ultrastructure of lignin carbohydrate complexes in softwoods. *Int J Biol Macromol* 62:705-713.
